# What was the global burden of kidney cancer attributable to high body mass index from 1990 to 2019? There existed some points noteworthy

**DOI:** 10.3389/fnut.2024.1358017

**Published:** 2024-06-05

**Authors:** Xue Yao, Xiao-yan Luo, Yang-hao Tai, Kang Wang, Ji-wen Shang

**Affiliations:** ^1^Department of Ambulance Surgery, Shanxi Bethune Hospital, Shanxi Academy of Medical Science, Tongji Shanxi Hospital, Third Hospital of Shanxi Medical University, Taiyuan, China; ^2^Department of Urology, Shanxi Bethune Hospital, Shanxi Academy of Medical Science, Tongji Shanxi Hospital, Third Hospital of Shanxi Medical University, Taiyuan, China

**Keywords:** GBD 2019, mortality, disability-adjusted life year, kidney cancer, high body mass index

## Abstract

**Purpose:**

With the prevalence of high body mass index (HBMI) increasing over the past 30 years, it is essential to examine the impact of obesity on kidney cancer. This study aims to explore the attributable burden of kidney cancer associated with HBMI and its proportion at different levels.

**Methods and materials:**

The data used in this research were obtained from the Global Burden of Diseases Study 2019. We utilized DisMod-MR 2.1, a Bayesian meta-regression tool, to estimate the burden of kidney cancer attributable to HBMI, which was measured by age-standardized mortality rate (ASMR) and age-standardized disability-adjusted life years rate (ASDR). Correlation analysis was conducted by the Spearman rank order correlation method. The temporal trends were analyzed by estimating the estimated annual percentage change (EAPC).

**Results:**

Globally in 2019, there were a total of 31.7 thousand deaths and 751.89 thousand disability-adjusted life years (DALYs) attributable to kidney cancer caused by HBMI, increased by 183.1 and 164%, respectively. Over the period from 1990 to 2019, the burden of kidney cancer attributable to HBMI increased in all regions, with the most significant increases occurring in Low-middle socio-demographic index (SDI) and Low SDI regions. At the national level, countries with lower SDI had lower ASMR and ASDR compared to developed nations. However, the EAPC values, which indicate the rate of increase, were significantly higher in these countries than in developed nations. Furthermore, across all years from 1990 to 2019, males experienced a greater and more rapidly increasing burden of kidney cancer attributable to HBMI than females.

**Conclusion:**

As the population grows and dietary patterns shift, the burden of kidney cancer attributable to HBMI is expected to become even more severe. Males and developed regions have borne a heavier burden from 1990 to 2019. However, the EAPC values for both ASMR and ASDR were higher in males but not in regions with higher SDI values.

## 1 Introduction

In this century, cancer is emerging as a significant public health issue and is poised to surpass cardiovascular disease as the leading cause of premature deaths attributable to non-communicable diseases worldwide. According to previous research, it was estimated that there were around 19.3 million new cases of cancer and 10.0 million cancer-related deaths in 2020 (1, 2). The global increase in population and the aging of populations have contributed to the rapid rise in the incidence and mortality of kidney cancer, which is a common genitourinary malignancy with poor prognosis. According to the latest data from the International Agency for Research on Cancer, kidney cancer is ranked ninth among males and sixteenth among females in terms of incidence (2, 3). In general, the incidence of kidney cancer is higher in males than in females, with a reported male-to-female ratio of approximately 1.5:1. Additionally, there is a positive association between the incidence rate of kidney cancer and the socio-demographic index (SDI), indicating that the incidence is generally higher in developed countries compared to developing countries (4, 5), which is consistent with our previous study (6).

In previous decades, smoking had long been considered the primary risk factor for kidney cancer. A previous study reported that smoking increased the risk of kidney cancer by 50% in males and 20% in females (7). However, our recent study has revealed a significant shift in the leading risk factor for kidney cancer. High body mass index (HBMI) has now surpassed smoking as the primary risk factor for both sexes and this trend is expected to continue in the future, furthermore, HBMI remains the most significant risk factor for kidney cancer among females worldwide in the past 30 years (6). HBMI is a pervasive issue in today’s society and is associated with various diseases, including ischemic heart disease, hepatocellular carcinoma, and hypertension (8–10), which not only poses a significant health risk but also places a substantial burden on financial and human resources. Addressing the impact of HBMI is crucial to alleviate the burden in both individuals and societies.

Understanding the burden of kidney cancer attributed to HBMI is crucial to developing effective preventative measures for this disease in the future. Previous studies on kidney cancer have mainly focused on describing the overall disease burden caused by all risk factors, offering only brief mentions of kidney cancer-associated risk factors, however falling short in specifically pinpointing the burden specifically attributable to HBMI and providing precise preventative measures to mitigate the burden stemming from HBMI (6, 11, 12). Therefore, we analyze data from Global Burden of Diseases Study 2019 (GBD 2019), combining with factors such as year, age, sex, and SDI, to examine the current trends in global, regional, and national mortality and disability-adjusted life years (DALYs) of kidney cancer attributable to HBMI. By assessing the changing burden of this disease, our study aims to provide valuable insights for medical professionals and policymakers, facilitating informed decision-making and effective management strategies.

## 2 Materials and methods

### 2.1 Data resource and disease definition

We utilized input data extracted from the GBD 2019 database (available at http://ghdx.healthdata.org/gbd-results-tool) to estimate the burden of kidney cancer. Clinical data sources for kidney cancer included hospital records, emergency department records, insurance claims, surveys, and vital registration systems worldwide was recorded in GBD 2019. The methodology for data inputting, mortality estimation, and modeling for GBD 2019 has been comprehensively demonstrated in previously published research articles (13, 14). Our research focuses on the burden of kidney cancer attributable to HBMI from 1990 to 2019 in 204 countries and territories. The definition of kidney cancer is based on the International Statistical Classification of Diseases and Related Health Problems, 10th Revision (ICD-10), with corresponding codes C64-C65.9, D30.0-D30.1, and D41.0-D41.1 (13). Furthermore, in accordance with the parent GBD risk factor study, HBMI was defined as a body-mass index (BMI) greater than 20–25 kg/m^2^ for adults aged 20 and above, while overweight and obese were corresponded to a BMI greater than 25 and 30 kg/m^2^, respectively (14).

### 2.2 Socio-demographic index

The burden of kidney cancer attributable to HBMI was calculated in relation to the level of development at the country level, as constrained by the SDI (15, 16), which is a composite indicator that combines three separate indicators: lag-distributed income *per capita*, average educational attainment for individuals aged 15 years and older, and the total fertility rate among individuals aged younger than 25 years. Based on this criteria, the 204 countries and territories were classified into five groups based on their SDI values: low SDI (<0.45), low-middle SDI (≥0.45 and < 0.61), middle SDI (≥0.61 and < 0.69), high-middle SDI (≥0.69 and < 0.80), and high SDI (≥0.80).

### 2.3 Risk factors

The risk factors in GBD 2019 were estimated on the basis of a comparative risk assessment framework including six steps. Firstly, the GBD risk factor estimation identified risk-outcome pairs, and only those outcomes that met the World Cancer Research Fund’s criteria of convincing or plausible evidence were included (14). Secondly, the relative risk (RR), which was considered a function of exposure for each risk-outcome pair, was estimated. To obtain the RR estimation, the GBD study conducted meta-analyses of RRs based on published systematic reviews, with 81 new systematic reviews added in GBD 2019. Thirdly, to estimate the distribution of risk exposure, household surveys, censuses, published studies, and governmental data were examined to determine the mean levels of risk exposure. Fourthly, the theoretical minimum risk exposure level (TMREL) was determined. In GBD 2019, the TMREL was set at 20–25 kg/m^2^ for adults aged 20 and above, based on the body mass index level associated with the lowest risk of all-cause mortality in prospective cohort studies. For children aged 2–19, the TMREL was defined as “normal weight,” which corresponded to not being overweight or obese according to IOTF cutoffs. Fifthly, the GBD study measured the population attributable fraction (PAF) and attributable burden. PAF was defined as the percentage of disease burden that could be reduced if the TMREL exposure to a specific risk factor were achieved, and it was calculated using the following formula: $PAF=\frac{\int_{x=l}^{m}RR\left(x\right)dx-RR\left(x\right)\mathrm{TRMEL}}{\int_{x=l}^{m}RR\left(x\right)P\left(x\right)dx}$ , where l represented the minimum exposure level and m the maximum exposure level. Finally, the PAF and attributable burden for combinations of risk factors were estimated. The methodology for these steps had been systematically demonstrated in previous GBD research (14).

### 2.4 Statistical analysis

In the current study, the age-standardized mortality rate (ASMR) and age-standardized disability-adjusted life years rate (ASDR), along with their corresponding 95% uncertainty intervals (95%UI), were used to evaluate and compare the mortality and DALYs of kidney cancer at both regional and national levels. In the GBD 2019, age-standardized rates are typically calculated using the 2019 version of the World Health Organization World Standard Population. This population structure, based on global age distribution patterns, represents a standardized demographic profile. The method entails defining age groups and assigning weights based on observed global age distribution. Age groups span from 0–4 to 95 years and above, each representing a population segment. Weights reflect the proportion of individuals in each age group. By applying population-specific rates to this standard structure, age-standardized rates are calculated, removing age distribution discrepancies when comparing disease burdens across populations (13). The study utilized age-standardized rates (ASRs) to calculate the estimated annual percentage change (EAPC) in both the ASMR and ASDR from 1990 to 2019 to investigate trends over time and presented the EAPC values with a 95% confidence interval (95%CI). The EAPC was estimated using the assumption of a linear relationship between ASRs and time, represented by the following formula: *y*Hart = *a* + *bx* + *e*. In this model, *y* = log10 (ASR), *x* = calendar year, and *b* represents the regression coefficient. The EAPC was calculated as EAPC = 100 * (*e*^*b*−1) based on this model. To determine trends in ASMR and ASDR, a lower boundary of 95% CI for EAPC above zero indicates an upward trend, and vice versa. Gaussian process regression with a Loess smoother was used to estimate the expected values of ASMR and ASDR within each SDI unit. Spearman’s rank order correlation was utilized to assess the correlation between the SDI and ASMR and ASDR. A *p* value <0.05 was considered statistically significant, and all statistical analysis was performed using R software (version 4.1.0; https://cran.r-project.org).

## 3 Results

### 3.1 Global, regional, and national burden of kidney cancer attributable to HBMI from 1990 to 2019

**Figure 1:**
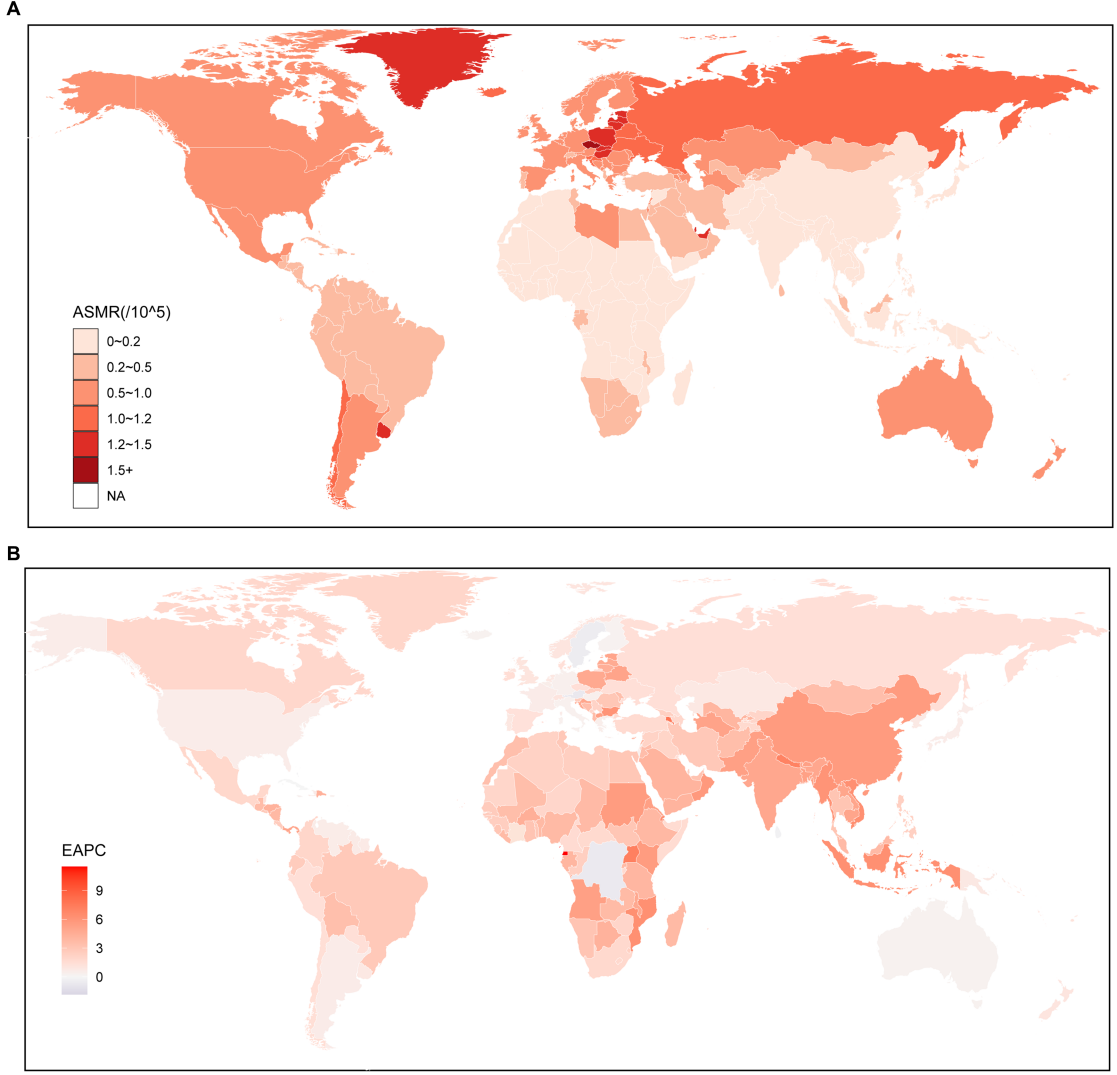
Global age standardized mortality rate of kidney cancer attributable to high body mass index. **(A)** The all-cause ASMR per 100,000 associated with kidney cancer attributable to high body mass index, for both sexes in 204 countries and territories in 2019. **(B)** The EAPC of ASMR of kidney cancer attributable to high body mass index, for both sexes from 1990 to 2019, in 204 countries and territories. ASMR, Age standardized mortality rate; EAPC, Estimated annual percentage change.

At the global level, the number of deaths of kidney cancer attributable to HBMI increased significantly from 11.20 (95% UI: 6.18–17.48) thousand in 1990 to 31.70 (95% UI: 18.43–47.28) thousand in 2019, which increased by 183% (95% UI: 159–219%). At the regional level, the highest ASMR was observed in Central Europe (1.2; 95% UI: 0.74–1.73), followed by Eastern Europe (1.12; 95% UI: 0.68–1.62) and Southern Latin America (1.06; 95% UI: 0.58–1.62). Conversely, the lowest ASMR was found in South Asia (0.08; 95% UI: 0.04–0.13) (Supplementary Table S1). At the national level, countries with high ASMR were predominantly located in Central and Western Europe, as well as North America. Notably, the country with the highest ASMR was Czechia (1.79; 95% UI: 1.05–2.67), followed by Estonia (1.55; 95% UI: 0.91–2.35). In 2019, countries in East Asia, Southeastern Asia, and Africa generally experienced lower ASMR, except for the United Arab Emirates (1.5; 95% UI: 0.44–2.83). Among these countries, Somalia had the lowest ASMR (0.03; 95% UI: 0–0.09), followed by Bangladesh (0.04; 95% UI: 0.01–0.08) and Papua New Guinea (0.04; 95% UI: 0.01–0.09) (Figure 1A; Supplementary Table S3).

To further investigate the impact of HBMI on the burden of kidney cancer, an analysis was conducted to examine the trends from 1990 to 2019. The findings indicated similar changing patterns for both sexes, males and females, at the global level. Notably, there was a significant increase in the ASMR of kidney cancer attributable to HBMI, particularly among males (1.41; 95% CI: 1.29–1.53) (Supplementary Table S1). From 1990 to 2019, there was a notable increase in the ASMR for kidney cancer attributed to HBMI across all regions. The largest increase was observed in East Asia (5.52; 95% CI: 5.08–5.97), followed by South Asia (4.88; 95% CI: 4.67–5.09). Conversely, the smallest increase was found in Oceania (0.48; 95% CI: 0.18–0.78). Interestingly, while regions with higher SDI values were associated with higher ASMR, the EAPC for these regions were relatively lower compared to other regions (Supplementary Table S1). Similar to the regional level, the majority of countries experienced an upward trend in the ASMR for kidney cancer attributed to HBMO from 1990 to 2019, among the countries with an increased ASMR over the same period, the highest increase was seen in Armenia (7.74; 95% CI: 6.76–8.73), closely followed by Uganda (7.2; 95% CI: 6.91–7.49) and Nepal (7.04; 95% CI: 6.81–7.27). However, there were a few exceptions, including Trinidad and Tobago, Bermuda, Saint Kitts and Nevis, Austria, Democratic Republic of the Congo, Saint Lucia, Barbados, and Northern Mariana Islands. Among these countries, the most significant decrease was observed in Trinidad and Tobago (−1.83; 95% CI: −2.47 to −1.18) (Figure 1B; Supplementary Table S3).

**Figure 2:**
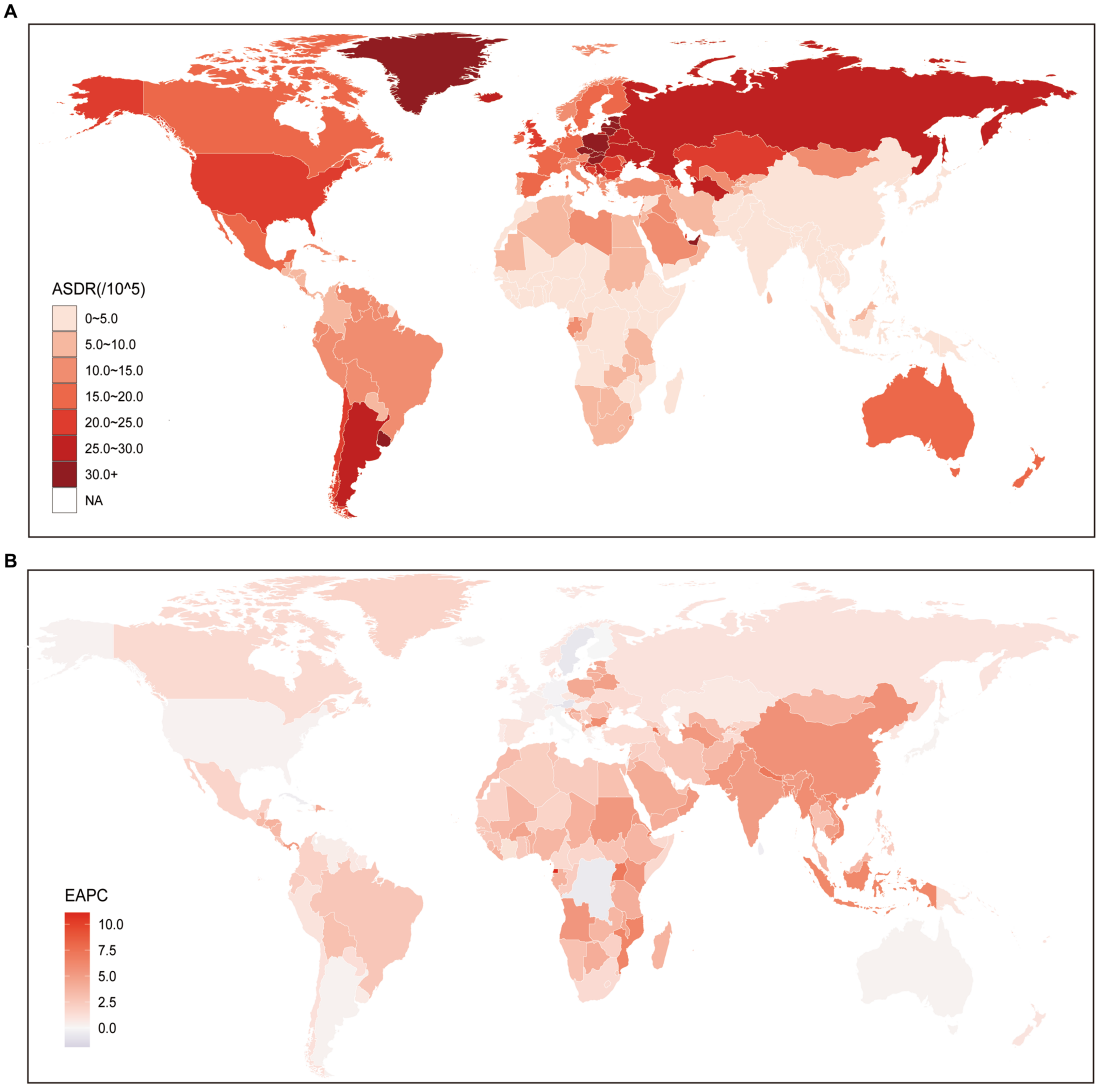
Global age standardized DALYs rate of kidney cancer attributable to high body mass index. **(A)** The all-cause ASDR per 100,000 associated with kidney cancer attributable to high body mass index, for both sexes in 204 countries and territories in 2019. **(B)** The EAPC of ASDR of kidney cancer attributable to high body mass index, for both sexes from 1990 to 2019, in 204 countries and territories. DALYs, Disease adjusted life year; ASDR, Age standardized DALYs rate; and EAPC, Estimated annual percentage change.

Globally, the number of DALYs attributed to kidney cancer attributable to HBMI increased from 285.06 thousand (95% UI: 156.91–442.28) in 1990 to 751.89 thousand (95% UI: 443.68–1114.6) in 2019, increased by 164% (95% UI: 140–201%). At the regional level, it is notable that regions with higher SDI face a more significant burden of ASDR for kidney cancer. Among these regions, Central Europe exhibited the highest ASDR (28.87; 95% UI: 17.67–41.31), followed by other high-income regions such as Eastern Europe (28.78; 95% UI: 17.25–41.54), High-income North America (22.55; 95% UI: 13.86–31.32), and Australasia (19.61; 95% UI: 11.81–28.33). Conversely, underdeveloped regions generally displayed lower ASDRs when compared to the aforementioned high SDI regions. The lowest ASDR was observed in South Asia (2.15; 95% UI: 1.16–3.45), followed by Central Sub-Saharan Africa (2.4; 95% UI: 1.15–4.13) and Oceania (2.44; 95% UI: 1.25–3.98). However, it is worth noting a significant point: Southern Latin America, a region primarily composed of developing countries, is facing a remarkably high ASDR for kidney cancer (25.9; 95% UI: 14.21–39.47), which even surpasses that of High-income North America and Australasia (Supplementary Table S2). The trend of ASDR at the national level was similar with that at the region level, developed countries were facing a heavier burden of kidney cancer attributable to HBMI (Figure 2A; Supplementary Table S4). More specifically, Czechia (40.4; 95% UI: 23.51–60.07) was facing the highest ASDR of kidney cancer attributable to HBMI, the following three countries were United Arab Emirates (39.6; 95% UI: 12.26–74.67), Greenland (35.47; 95% UI: 19.57–55.01), and Estonia (34.86; 95% UI: 20.66–53.86). On the contrary, the lowest ASDR was seen in Somalia (0.78; 95% UI: 0.13–2.23), and the following three countries were Bangladesh (1.08; 95% UI: 0.43–2.1), Democratic People’s Republic of Korea (1.16; 95% UI: 0.22–2.93), and Papua New Guinea (1.2; 95% UI: 0.43–2.5), which were characterized by undeveloped finance and the leakage of food (Figure 2A; Supplementary Table S4).

The analysis of the EAPC of the ASDR of kidney cancer attributed to HBMI from 1990 to 2019 revealed consistently increasing trends. At the global level, both sexes exhibited a continuously increasing trend during this period at 0.78 (95% CI: 0.69–0.88). However, it is worth noting that males (0.19; 95% CI: 0.11–0.27) experienced a faster increase in the ASDR of kidney cancer attributed to HBMI than females (Supplementary Table S2), which aligns with the changing trend of ASMR. While a general increasing trend in ASMR was observed globally, our results at the regional level revealed that Middle SDI, Low-middle SDI, and Low SDI regions exhibited significantly higher EAPC values compared to High SDI and High-middle SDI regions. Notably, the EAPC values were particularly higher in Low-middle SDI regions. Furthermore, our study identified the regions with the highest and lowest increases in EAPC of ASDR from 1990 to 2019. The region with the greatest increase was found to be Central Europe (5.58; 95% CI: 5.14–6.03), followed by South Asia (5.08; 95% CI: 4.89–5.26) and Eastern Sub-Saharan Africa (4.33; 95% CI: 4.09–4.58). Conversely, Australasia (0.3; 95% CI: 0.21–0.4), a High SDI region, exhibited the lowest growth in ASDR over the same period. Additionally, another High SDI region, High-income North America (0.3; 95% CI: 0.16–0.45), had a relatively similar EAPC value to that of Australasia (Supplementary Table S2). At the national level, we observed a decreasing trend in ASDR of kidney cancer attributed to HBMI in 18 countries, which were primarily located in regions with higher SDI, such as Central Europe. Among these countries, Trinidad and Tobago exhibited the lowest EAPC value (−1.83; 95% CI: −2.48 to −1.17), followed by Bermuda (−1.77; 95% CI: −2.3 to −1.24) and Saint Kitts and Nevis (−1.69; 95% CI: −2.3 to −1.07). In contrast, the countries with the highest increase in ASDR were Cabo Verde, with an EAPC value of 7.31 (95% CI: 6.63–7.99), followed by Armenia (7.29; 95% CI: 6.35–8.23), Uganda (7.13; 95% CI: 6.84–7.42), and Nepal (7.11; 95% CI: 6.83–7.39) (Figure 2B; Supplementary Table S4).

### 3.2 Correlation between the burden of kidney cancer attributable to HBMI with SDI

**Figure 3:**
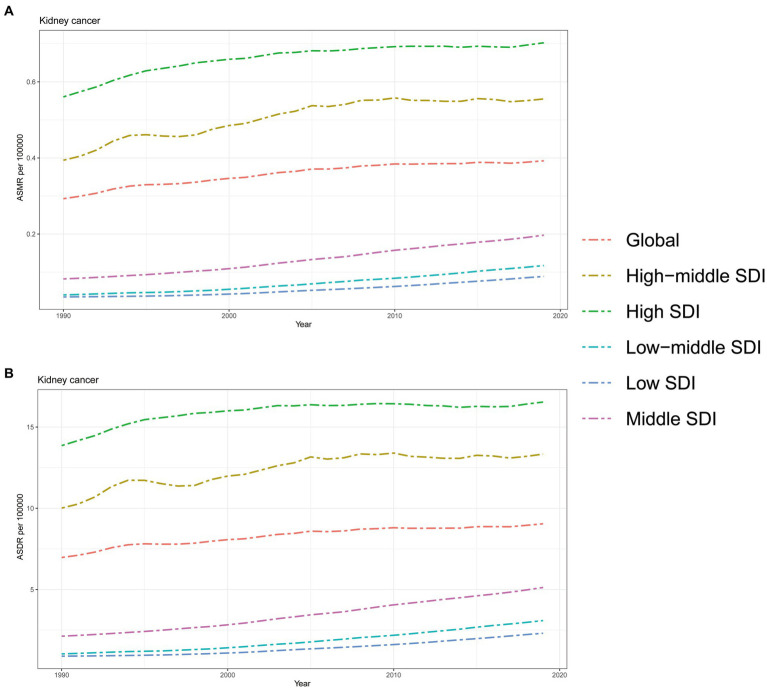
The burden of kidney cancer attributable to high body mass index by SDI. **(A)** The ASMR and **(B)** ASDR of kidney cancer attributable to high body mass index in different SDI regions from 1990 to 2019. Results are showed for both sexes worldwide. ASMR, Age standardized mortality rate; DALYs, Disease adjusted life year; ASDR, Age standardized DALYs rate; SDI, Sociodemographic index.

As depicted in Figure 3, the ASMR and ASDR of kidney cancer attributed to HBMI displayed a consistent upward trend from 1990 to 2019, both globally and across all GBD regions. It is worth noting that regions with a higher SDI, such as High SDI and High-middle SDI regions, faced a greater burden of ASMR and ASDR compared to the global average. Conversely, regions with lower SDI values experienced a relatively lower burden, with ASMR and ASDR rates below the global average. However, it is important to highlight that while regions with higher SDI, such as High SDI and High-middle SDI regions, bore a heavier burden compared to regions with lower SDI values; the rate of increase in both ASMR and ASDR was consistently higher in Middle SDI, Low-middle SDI, and Low SDI regions. In fact, the increasing speeds in these lower SDI regions surpassed those in the higher SDI regions over the years while the rate of increase in High SDI and High-middle SDI regions showed a gradual decline during this period (Figure 3).

**Figure 4:**
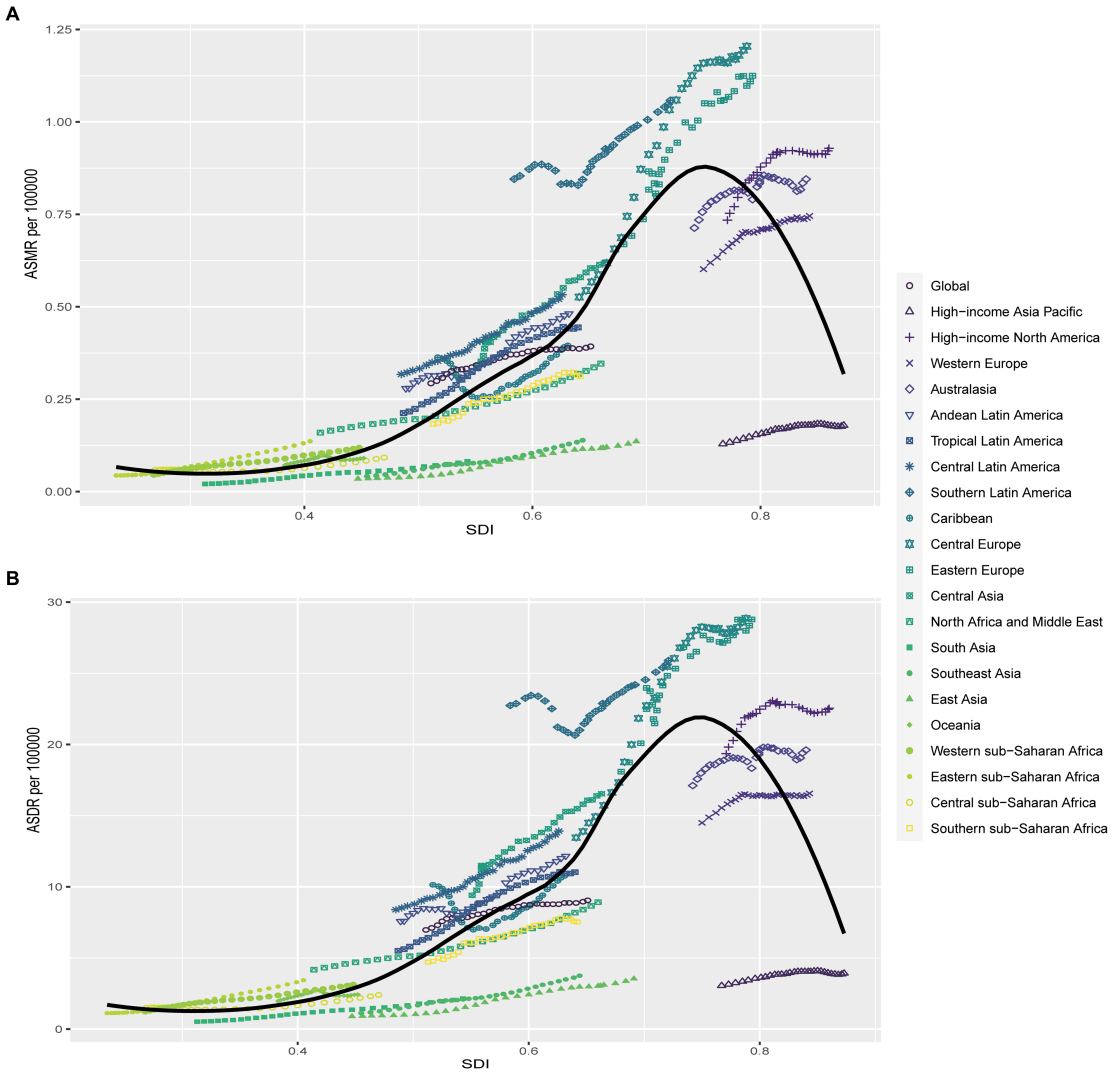
Correlations of ASMR as well as ASDR of kidney cancer attributable to high body mass index and SDI at the regional level. The ASMR **(A)** and ASDR **(B)** of kidney cancer attributable to high body mass index and SDI at the regional level in 21 regions from 1990 to 2019. ASMR, Age standardized mortality rate; DALYs, Disease adjusted life year; ASDR, Age standardized DALYs rate.

Correlation analysis was conducted to explore the relationship between SDI and regions as well as nations in relation to ASMR and ASDR of kidney cancer attributed to HBMI. Our findings revealed that higher SDI levels were generally associated with higher ASMR and ASDR rates, however, no linear associations were found for both ASMR and ASDR (Figure 4; Supplementary Figure S1). At the regional level, Central Europe, Eastern Europe, and Southern Latin America exhibited higher ASMR than anticipated based on their SDI values during the observed period. Conversely, East Asia, Southeast Asia, South Asia, and High-income Asia Pacific regions experienced lower ASMR rates than expected given their SDI levels (Figure 4A). The same phenomenon was also seen in the results of ASDR for these regions (Figure 4B). Comparing the observed ASMR and ASDR with the expected levels based on SDI values at the national level, the results were consistent with those at the regional level. While the Czech Republic had the highest ASMR and ASDR rates in 2019, United Arab Emirates showed the largest disparity in both ASMR and ASDR for kidney cancer attributed to HBMI according to our study (Supplementary Figure S1).

### 3.3 Age and sex patterns

**Figure 5:**
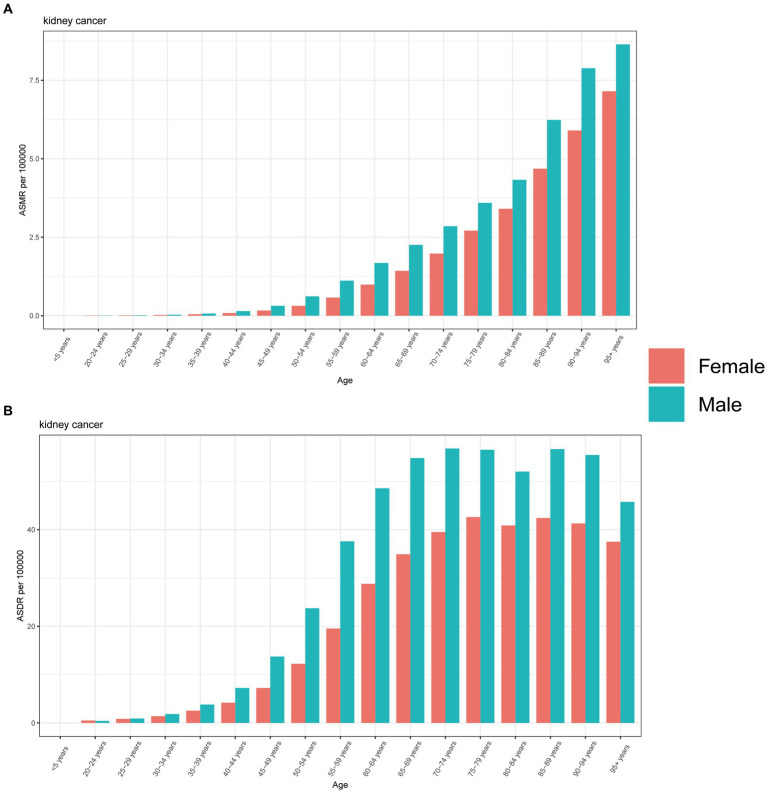
The burden of kidney cancer attributable to high body mass index by age and sex. The all-cause ASMR **(A)** and ASDR **(B)** of kidney cancer attributable to high body mass index worldwide in different age groups. ASMR, Age standardized mortality rate; DALYs, Disease adjusted life year; ASDR, Age standardized DALYs rate.

In 2019, males exhibited higher ASMR across all age groups, except for individuals aged 20–24 years. Furthermore, this disparity increased with age, with the largest difference observed in the 95+ age group (Figure 5A). The same pattern was also found in the analysis of ASDR of kidney cancer attributable to HBMI, while the largest separation was observed among individuals aged 65–69 years, and this difference decreased with age. Notably, another peak in ASDR was observed among those aged 85–94 years, followed by a sharp decline among those aged 95+ years (Figure 5B).

**Figure 6:**
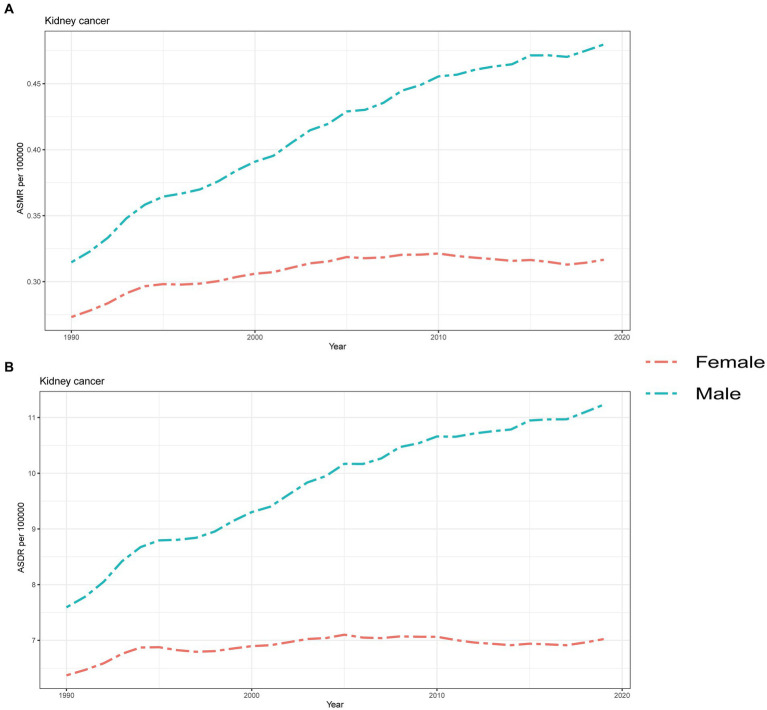
Global attributable burden of kidney cancer attributable to high body mass index by sex. The age-standard ASMR **(A)** and ASDR **(B)** of kidney cancer attributable to high body mass index by sex from 1990 to 2019. ASMR, Age standardized mortality rate; DALYs, Disease adjusted life year; ASDR, Age standardized DALYs rate.

From 1990 to 2019, males consistently experienced a higher burden of kidney cancer attributed to HBMI, as indicated by both ASMR and ASDR. Moreover, these disparities gradually increased over the years (Figure 6). On the other hand, the ASMR for females showed a slow increase from 1990 to 2005, followed by fluctuations within a small range around the baseline (Figure 6A). A similar pattern was observed in the ASDR for females; with the rates stabilizing after 1994 and fluctuating for the next 26 years (Figure 6B). In contrast, both ASMR and ASDR for males consistently rose over the 30-year period, although there was a noticeable decrease in the rate of increase starting in 1994 (Figure 6).

The ratio of male to female mortality and DALYs rates exhibited a significant difference, although an overall increasing trend was observed during this period (Supplementary Figure S2). The ratio of male to female DALYs rate steadily increased at a relatively stable pace over the course of 30 years (Supplementary Figure S2B). However, the growth of the ratio of male to female mortality was more erratic, with seven instances of decline occurring in 1991, 1993, 1995, 1997, 1999, 2004, and 2016, respectively (Supplementary Figure S2A).

## 4 Discussion

Although kidney cancer had a relatively lower incidence compared to bladder cancer and prostate cancer, there has been a significant increase in its incidence over the past 30 years. Interestingly, contrary to common knowledge, HBMI has now surpassed smoking as the leading risk factor for kidney cancer mortality and DALYs rate (6, 11, 17). In this study, we conducted a comprehensive analysis of the burden of kidney cancer attributable to HBMI and its associations with SDI, age, and year. Our findings showed that globally, the ASMR and ASDR of kidney cancer attributable to HBMI remained substantial and increased significantly from 1990 to 2019, particularly in males. At the regional level, due to higher incidences in developed regions such as Central Europe and Western Europe with higher SDI values, the ASDRs and ASMRs in these regions were the highest among all regions. However, the rate of increase, as reflected by the EAPC value, was noticeably lower in these regions compared to those with lower SDI values. Similar trends were also observed at the national level; countries located in High SDI and High-middle SDI regions have a higher burden of kidney cancer attributable to HBMI than developing countries. Females had lower ASMR and ASDR than males, and the gender gap in ASDR peaked in the 65–69 age group while it gradually increased with age for ASMR.

The increasing trend in the incidence of kidney cancer across nearly all regions and nations can be partly attributed to the rapidly growing global population and increased life expectancy from 1990 to 2019 (6, 17). Furthermore, the advancement and widespread use of abdominal cross-sectional imaging technology have also led to a greater number of incidental diagnoses of kidney cancer in all countries, consequently contributing to the rise in kidney cancer incidence (6, 18), while the increased incidence ultimately results in higher ASMR and ASDR. The change in dietary patterns, particularly the increased availability, affordability, and accessibility of food, along with the widespread use of international beverage trades, played a significant role in the rise of diseases associated with HBMI, including kidney cancer (19, 20). At the region level, regions with higher SDI values were facing a severer burden of kidney cancer attributable to HBMI, which might as a result of high proportions of overweight people in regions with higher SDI value (21). Moreover, increased health awareness, sufficient medical resources, and favorable economic conditions have led to a rise in the incidental detection of abnormal kidney structures during abdominal imaging for other diseases or routine physical examinations in these regions (22). Furthermore, as the globally recognized gold standard surgical method for treating kidney cancer, this surgery ensures the preservation of renal function to the greatest extent possible while also ensuring effective treatment and longer cancer-specific survival rates (23), thus leading to the increase of ASDR. It is worth noting that while regions with higher SDI values had both high ASMR and ASDR; regions with lower SDI had considerably higher EAPC values compared to higher-income regions from 1990 to 2019. On the one hand, despite the gradually improving living conditions and progressively abundant food supply, the lack of awareness about HBMI in these lower SDI regions has led to an increase in the burden of diseases related to HBMI in these areas (21). On the other hand, regions with higher SDI values exhibiting higher ASMR and ASDR values may experience lower EAPC values because the burden of kidney cancer attributable to HBMI are already sever. Additionally, a declining birth rate could also contribute to lower EAPC values, as kidney cancer predominantly affects older individuals. Another noteworthy point is that the ASMR and ASDR in High-income Asia Pacific countries were considerably lower than the expected values based on their association with SDI although the EAPC values of both ASMR and ASDR in High-income Asia Pacific were similar to other regions with high SDI values. On the other hand, the ASMR and ASDR in Southern Latin America were significantly higher than expected, but the EAPC value in this region was comparable to other regions with similar SDI values, which aligns with the previous research findings on the trends in body mass index (24). In our research, Czechia demonstrated the highest values for both ASMR and ASDR at the national level, which might be explained by the high percentage of overweight and obese individuals in Czechia (25, 26). A similar trend was observed at the national level, where 17 countries showed a decrease in ASMR and 19 countries showed a decrease in ASDR from 1990 to 2019, while these countries were predominantly located in High SDI and High-middle SDI regions, such as Central Europe. In general, regions with higher SDI values are characterized by ample medical resources and the population in these regions willing to undergo routine physical exams, supported by relatively prosperous economic conditions and effective health advocacy and education, contributing to a general decrease in the burden of kidney cancer attributable to HBMI over the past 30 years (27), which on the other hand highlights the significance of a country’s level of development in influencing the burden of kidney cancer attributable to HBMI.

Our global study revealed a persistent gender gap, with males having significantly higher ASMR and ASDR than females. Furthermore, this gap has been increasing in recent years due to the higher EAPC values of ASMR and ASDR attributed to HBMI in males from 1990 to 2019. Previous research has shown that smoking, rather than HBMI, was the most significant risk factor for ASMR and ASDR in male kidney cancer cases worldwide since 1990 (6, 28), however, our study revealed that the burden of kidney cancer attributed to HBMI is more severe in males compared to females (6). From 1990 to 2019, there has been a gradual increase in the ratio of male to female mortality and the ratio of male to female DALYs rate, which indicates a greater burden of kidney cancer in males. Additionally, it has been demonstrated that the frequency ratio between males and females is greater than 1 for all cancers, except for sex-specific malignancies such as prostate cancer, breast cancer, and thyroid cancer (29). This result might be explained by the different method of white adipose tissue deposition, Lemieux et al. (30) reported that pre-menopausal females tend to store white adipose tissue in their femoral and gluteal lower body depots, while males tend to develop visceral fat mass. Lower body depots have been shown to have better lipid handling properties and a healthier metabolic profile (31). Furthermore, research has shown that almost 50% of abdominal fat in females is stored in the central compartment, compared to 98% in males and increased visceral adiposity is associated with obesity and often linked to the progression of metabolic-related conditions (32). Therefore, males have been found to have a higher BMI compared to females, which is considered as a major risk factor for various non-communicable diseases and has been proven to be associated with an increased risk of several types of cancer according to numerous studies, therefore has also received significant attention in recent years (33–35). Our findings also indicate that the burden of kidney cancer attributed to HBMI increases with age, which may be partly due to cellular senescence and mitochondrial dysfunction. Previous research has clearly shown that DNA repair competence deteriorates with age and that DNA damage accumulates in the genome, leading to cellular senescence (36, 37). Senescent cells, however, had the possibility to generate tumorigenesis by secreting inflammatory cytokines (38). Previous articles had revealed the accumulation of point mutations and deletions in mitochondrial genome during aging (39, 40), thus leading to many age-related diseases such as Alzheimer’s disease, Parkinson’s disease, and cancer (41).

According to the European Urology Guideline, obesity has been identified as one of significant lifestyle factors for kidney cancer [hazard ratio (HR): 1.71] (42), and it is reported that the association between kidney cancer and obesity is linear, with a 4% increase in the risk of kidney cancer for every one-point increment in BMI (43). Furthermore, it is strongly recommended to enhance physical activity and reduce weight as primary preventive measures to decrease the risk of kidney cancer and the most effective prophylaxis involves avoiding cigarette smoking and reducing obesity according to this guideline (42). However, although there is a growing interest from both patients and clinicians in kidney cancer screening programs, it not recommand to perform routinely screen among any population for primary kidney cancer due to the limitation of screening measures, such as unambiguous biomarkers in urinay and serum, the cost and radiation dose of computed tomography and the operator dependant of ultrasound (42).

To our knowledge, this is the first study to examine the burden of kidney cancer associated with HBMI from 1990 to 2019 basing on the GBD 2019 dataset. Considering the significant impact of HBMI on cancer incidence, we conducted this research to raise awareness about the importance of weight loss among overweight and obese individuals, particularly males. Our research also has some internal limitations that are common for all GBD burden estimates (13, 14). The primary limitation of this study lies in the availability and completeness of the source data, which mainly consisted of prospective observational studies. Furthermore, countries with lower SDI values often face challenges in collecting accurate and comprehensive health data, which can impact the reliability and completeness of their contributions to GBD studies. First of all, many developing countries lack robust health information systems, making it difficult to gather timely and accurate data on disease prevalence, mortality rates, and risk factors; secondly, limited access to healthcare facilities and diagnostic services can lead to underreporting or misclassification of diseases and causes of death, resulting in gaps and inaccuracies in the data in these countries; thirdly, these countries may have fewer resources allocated to health research, hindering their ability to conduct comprehensive studies and collect high-quality data. However, the GBD project is required to generate final estimates using statistical methods and predictive covariate values.

## 5 Conclusion

Over the past 3 decades, there has been a significant increase in the burden of kidney cancer attributable to HBMI worldwide, paralleling the rising prevalence of HBMI. At the regional and national levels, high-income regions and developed countries have experienced a higher burden of cancer attributable to HBMI, although with a lower EAPC values in incidence rates, particularly in Central Europe and East Europe (excluding high-income Asia Pacific). Additionally, males have consistently exhibited higher values and annual percentage change in both ASMR and ASDR of kidney cancer attributable to HBMI compared to females over the 30-year period. Therefore, it is crucial to prioritize additional education on healthy lifestyles and diets for males in all countries, aiming to reduce the burden in regions and countries with higher SDI values and curtail the growth of burden in underdeveloped regions and countries.

## Data availability statement

The original contributions presented in the study are included in the article/Supplementary material, further inquiries can be directed to the corresponding author.

## Author contributions

XY: Conceptualization, Investigation, Software, Writing – original draft, Writing – review & editing. X-yL: Conceptualization, Writing – review & editing. Y-hT: Methodology, Writing – original draft. KW: Software, Writing – original draft. J-wS: Conceptualization, Investigation, Writing – original draft, Writing – review & editing.

## Supplementary material

SUPPLEMENTARY FIGURE S1 Correlations of ASMR as well as ASDR and SDI at the national level. The ASMR **(A)** as well as ASDR **(B)** of kidney cancer attributable to high body mass index and SDI at the regional level in 204 countries and territories from 1990 to 2019. ASMR, Age standardized mortality rate; DALYs, Disease adjusted life year; ASDR, Age standardized DALYs rate; and SDI, Sociodemographic index.

SUPPLEMENTARY FIGURE S2 Sex disparity in the global exposure and attributable burden of kidney cancer attributable to high body mass index in different years. Ratio of male to female ASMR **(A)** and ASDR **(B)** of kidney cancer attributable to high body mass index from 1990 to 2019. ASMR, Age standardized mortality rate; DALYs, Disease adjusted life year; and ASDR, Age standardized DALYs rate.
