# Supplementary figures and images for: What was the global burden of kidney cancer attributable to high body mass index from 1990 to 2019? There existed some points noteworthy
Source: Front Nutr. 2024 Jun 5;11:1358017. doi: 10.3389/fnut.2024.1358017 (PMC11188334; doi:10.3389/fnut.2024.1358017)

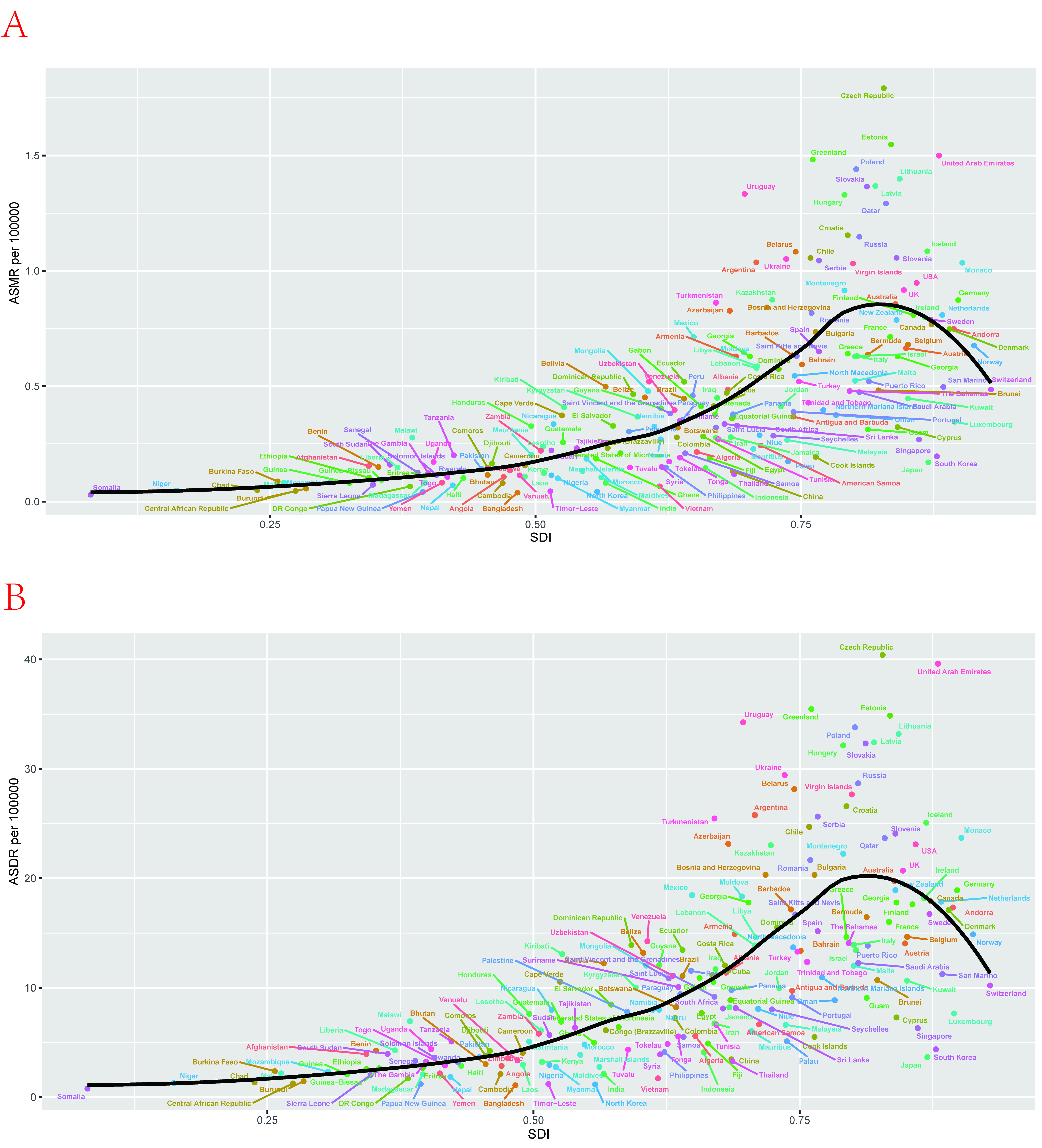

Supplement: SUPPLEMENTARY FIGURE S1 — Correlations of ASMR as well as ASDR and SDI at the national level. The ASMR (A) as well as ASDR (B) of kidney cancer attributable to high body mass index and SDI at the regional level in 204 countries and territories from 1990 to 2019. ASMR, Age standardized mortality rate; DALYs, Disease adjusted life year; ASDR, Age standardized DALYs rate; and SDI, Sociodemographic index. [file Image_1.TIF]

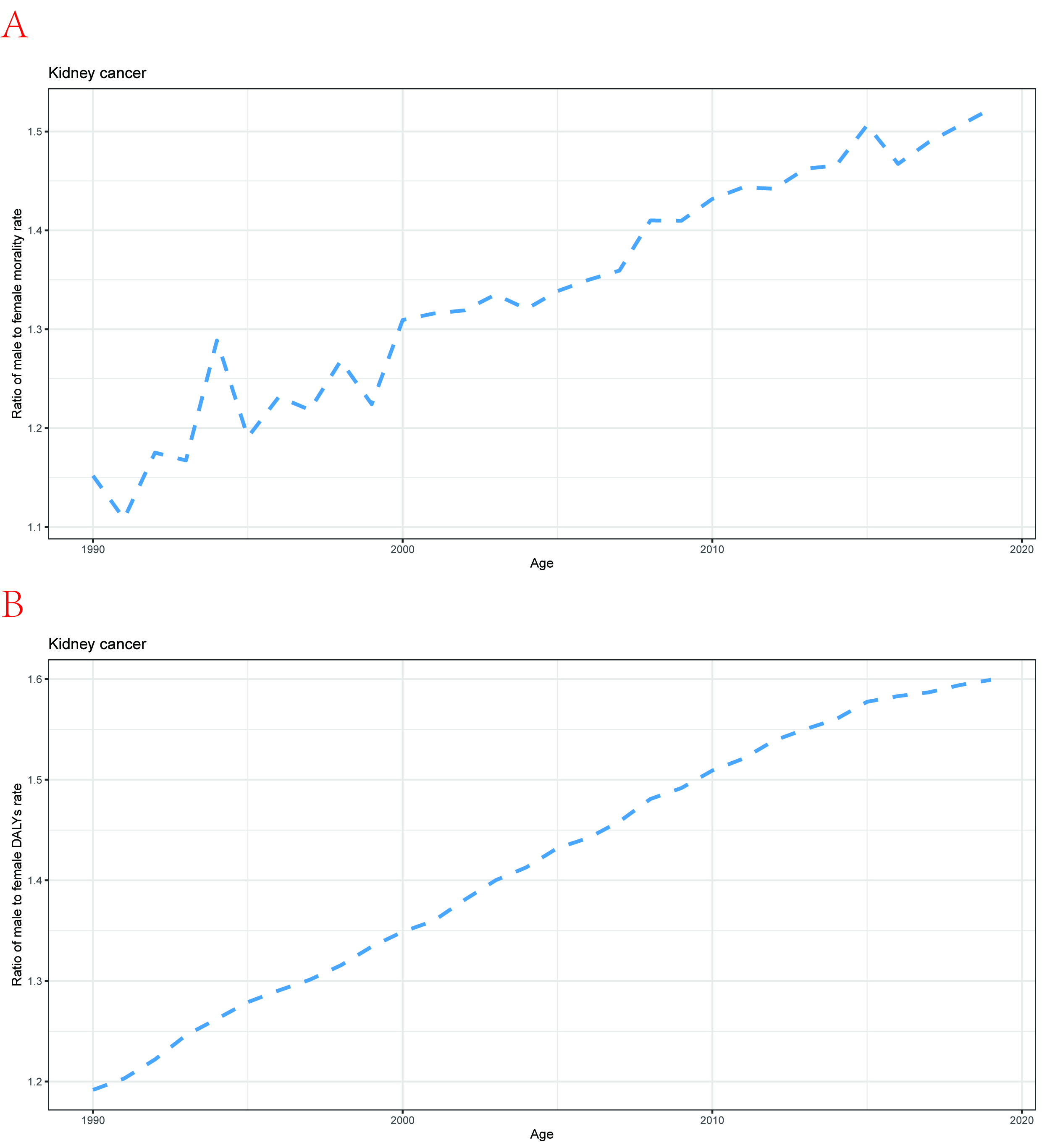

Supplement: SUPPLEMENTARY FIGURE S2 — Sex disparity in the global exposure and attributable burden of kidney cancer attributable to high body mass index in different years. Ratio of male to female ASMR (A) and ASDR (B) of kidney cancer attributable to high body mass index from 1990 to 2019. ASMR, Age standardized mortality rate; DALYs, Disease adjusted life year; and ASDR, Age standardized DALYs rate. [file Image_2.TIF]
