# Supplementary material for: What was the global burden of kidney cancer attributable to high body mass index from 1990 to 2019? There existed some points noteworthy
Source: Front Nutr. 2024 Jun 5;11:1358017. doi: 10.3389/fnut.2024.1358017 (PMC11188334; doi:10.3389/fnut.2024.1358017)
Supplement: Supplementary file 3 [file Table_1.docx]

Supplementary Table 1. Global and regional number of deaths and age-standardized morality of kidney cancer attributable to smoking for both sexes combined in 1990 and 2019, and EAPC of ASMR from 1990 to 2019

|  | Deaths number(×1000) in 1990 | Deaths number in 2019 | ASMR in 1990 | ASMR in 2019 | EAPC 1990-2019 |
| --- | --- | --- | --- | --- | --- |
| Global  Gender | \| 11.20  (6.18to17.48) \| \| --- \| | 31.70  (18.43to47.28) | 0.29 (0.16to0.46) | \| 0.39(0.23to0.56) \| \| --- \| | 0.94 (0.83 to 1.05) |
| Male | 5.50(2.60to9.20) | 17.83 (9.17to 28.16) | 0.31(0.15to0.53) | 0.48(0.25to0.76) | 1.41 (1.29 to 1.53) |
| Female  SDI | 5.70(3.27to5.76) | 13.87 (8.51to20.45) | 0.27(0.16to0.42) | 0.32(0.19to0.47) | 0.41 (0.31 to 0.52) |
| High SDI | 5.82(3.21to8.98) | 13.39(7.84to19.86) | 0.56(0.31to0.86) | 0.7(0.41to1.03) | 0.63 (0.51 to 0.75) |
| High-middle SDI | 4.22(2.39to6.48) | 11.34(6.6to16.88) | 0.39(0.22to0.61) | 0.56(0.32to0.83) | 1.11 (0.94 to 1.28) |
| Middle SDI | 0.83(0.41to1.42) | 4.9(2.76to7.6) | 0.08(0.04to0.14) | 0.2(0.11to0.31) | 3.24 (3.16 to 3.32) |
| Low-middle SDI | 0.24(0.1to0.45) | 1.6(0.88to2.54) | 0.04(0.02to0.08) | 0.12(0.06to0.19) | 3.94 (3.85 to 4.03) |
| Low SDI  Region | 0.08(0.03to0.17) | 0.46(0.24to0.76) | 0.03(0.01to0.07) | 0.09(0.05to0.15) | 3.51 (3.35 to 3.67) |
| Andean Latin America | 0.06(0.03to0.09) | 2.77(1.17to5) | 0.28(0.15to0.45) | 0.48(0.27to0.75) | 1.99 (1.81 to 2.17) |
| Australasia | 0.17(0.09to0.26) | 0.86(0.43to1.45) | 0.71(0.4to1.09) | 0.85(0.5to1.23) | 0.39 (0.27 to 0.51) |
| Caribbean | 0.1(0.05to0.15) | 0.85(0.31to1.61) | 0.36(0.21to0.55) | 0.39(0.23to0.61) | 0.55 (0.02 to 1.09) |
| Central Asia | 0.16(0.09to0.26) | 6.98(3.93to10.67) | 0.34(0.18to0.53) | 0.62(0.37to0.91) | 2.05 (1.94 to 2.16) |
| Central Europe | 0.78(0.47to1.15) | 3.9(2.35to5.6) | 0.53(0.31to0.77) | 1.2(0.74to1.73) | 2.98 (2.51 to 3.45) |
| Central Latin America | 0.27(0.15to0.42) | 0.88(0.48to1.35) | 0.32(0.17to0.49) | 0.53(0.3to0.8) | 1.81 (1.78 to 1.84) |
| Central Sub-Saharan Africa | 0.01(0.01to0.02) | 5.85(3.54to8.24) | 0.06(0.02to0.11) | 0.09(0.04to0.16) | 1.19 (0.68 to 1.69) |
| East Asia | 0.3(0.07to0.67) | 1.26(0.72to1.9) | 0.03(0.01to0.08) | 0.14(0.06to0.25) | 5.52 (5.08 to 5.97) |
| Eastern Europe | 1.9(1.13to2.82) | 0.42(0.25to0.61) | 0.67(0.4to1) | 1.12(0.68to1.62) | 1.65 (1.46 to 1.85) |
| Eastern Sub-Saharan Africa | 0.03(0.01to0.07) | 0.47(0.28to0.7) | 0.04(0.01to0.1) | 0.14(0.07to0.23) | 4.41 (4.16 to 4.66) |
| High-income Asia Pacific | 0.26(0.08to0.51) | 1.5(0.89to2.2) | 0.13(0.04to0.26) | 0.18(0.07to0.33) | 0.96 (0.73 to 1.19) |
| High-income North America | 2.53(1.41to3.79) | 2.59(1.58to3.71) | 0.73(0.41to1.1) | 0.93(0.57to1.3) | 0.64 (0.49 to 0.79) |
| North Africa and Middle East | 0.27(0.15to0.44) | 0.01(0to0.01) | 0.16(0.09to0.26) | 0.35(0.21to0.51) | 2.69 (2.62 to 2.77) |
| Oceania | 0(0to0) | 0.2(0.12to0.32) | 0.07(0.03to0.12) | 0.09(0.04to0.15) | 0.48 (0.18 to 0.78) |
| South Asia | 0.11(0.04to0.24) | 0.05(0.02to0.09) | 0.02(0.01to0.04) | 0.08(0.04to0.13) | 4.88 (4.67 to 5.09) |
| Southeast Asia | 0.11(0.04to0.22) | 0.27(0.15to0.42) | 0.04(0.02to0.08) | 0.14(0.07to0.24) | 3.86 (3.61 to 4.11) |
| Southern Latin America | 0.39(0.2to0.64) | 1.07(0.66to1.55) | 0.85(0.42to1.38) | 1.06(0.58to1.62) | 0.78 (0.64 to 0.92) |
| Southern Sub-Saharan Africa | 0.05(0.03to0.07) | 1.16(0.62to1.85) | 0.18(0.11to0.27) | 0.31(0.2to0.43) | 2.03 (1.85 to 2.2) |
| Tropical Latin America | 0.19(0.1to0.31) | 0.17(0.11to0.24) | 0.21(0.11to0.34) | 0.44(0.27to0.64) | 2.78 (2.59 to 2.98) |
| Western Europe | 3.45(1.88to5.42) | 0.23(0.12to0.36) | 0.6(0.33to0.95) | 0.75(0.42to1.13) | 0.58 (0.48 to 0.67) |
| Western Sub-Saharan Africa | 0.04(0.02to0.08) | 0.22(0.12to0.36) | 0.05(0.02to0.09) | 0.12(0.06to0.19) | 3.25 (3.18 to 3.32) |

ASMR, age-standard morality rate; EAPC, estimated annual percentage change.
