# Supplementary material for: What was the global burden of kidney cancer attributable to high body mass index from 1990 to 2019? There existed some points noteworthy
Source: Front Nutr. 2024 Jun 5;11:1358017. doi: 10.3389/fnut.2024.1358017 (PMC11188334; doi:10.3389/fnut.2024.1358017)
Supplement: Supplementary file 4 [file Table_2.docx]

Supplementary Table 2. Global and regional number of DALYs and age-standardized DALYs rate of kidney cancer attributable to smoking for both sexes combined in 1990 and 2019, and EAPC of ASMR from 1990 to 2019

|  | DALYs number (×1000) in 1990 | DALYs number (×1000) in 2019 | ASDR in 1990 | ASDR in 2019 | EAPC 1990-2019 |
| --- | --- | --- | --- | --- | --- |
| Global  Gender | \| 285.06  (156.91to442.28) \| \| --- \| | 751.89  (443.68to1114.6) | 6.97 (3.83to10.83) | \| 9.05(5.33to13.42) \| \| --- \| | 0.78 (0.69 to 0.88) |
| Male | \| 147.81(70.42to246.4) \| \| --- \| | \| 446.2(233.48to698.53) \| \| --- \| | \| 7.59(3.62to12.71) \| \| --- \| | \| 11.23(5.85to17.61) \| \| --- \| | 1.26 (1.14 to 1.37) |
| Female  SDI | 137.25(78.46to211.06) | 305.69(189.85to447.62) | 6.37(3.65to9.80) | 7.02(4.36to10.28) | 0.19 (0.11 to 0.27) |
| High SDI | 139.64(77.32to214.68) | 284.14(169.59to415.69) | 13.86(7.69to21.28) | 16.54(9.95to24.07) | 0.42 (0.31 to 0.54) |
| High-middle SDI | 111.36(63.25to170.07) | 272.11(158.46to405.21) | 10.01(5.67to15.31) | 13.33(7.74to19.85) | 0.85 (0.69 to 1.01) |
| Middle SDI | 24.5(12.12to41.41) | 136.1(77.77to211.16) | 2.13(1.05to3.61) | 5.12(2.93to7.98) | 3.23 (3.16 to 3.3) |
| Low-middle SDI | 7(2.95to13.06) | 45.6(25.51to71.87) | 1.04(0.44to1.95) | 3.09(1.72to4.87) | 4.01 (3.91 to 4.11) |
| Low SDI  Region | 2.38(0.85to4.87) | 13.53(7.11to21.98) | 0.9(0.32to1.84) | 2.31(1.21to3.77) | 3.55 (3.39 to 3.71) |
| Andean Latin America | 1.72(0.91to2.74) | 7.02(3.95to10.84) | 7.57(4.02to12.08) | 12.16(6.85to18.78) | 1.67 (1.46 to 1.87) |
| Australasia | 3.95(2.21to6.04) | 8.94(5.36to12.93) | 17.11(9.57to26.09) | 19.61(11.81to28.33) | 0.3 (0.21 to 0.4) |
| Caribbean | 2.77(1.59to4.22) | 5.57(3.22to8.58) | 10.14(5.83to15.43) | 10.77(6.23to16.61) | 0.42 (-0.13 to 0.97) |
| Central Asia | 4.74(2.54to7.4) | 14.04(8.36to20.82) | 9.4(5.09to14.74) | 16.54(9.9to24.44) | 1.87 (1.77 to 1.98) |
| Central Europe | 20.21(12.03to29.64) | 58.2(35.54to83.18) | 13.45(8to19.77) | 28.87(17.67to41.31) | 2.71 (2.24 to 3.18) |
| Central Latin America | 7.76(4.25to11.95) | 34.08(19.44to51.48) | 8.39(4.61to12.92) | 13.92(7.93to21.01) | 1.77 (1.73 to 1.8) |
| Central Sub-Saharan Africa | 0.39(0.16to0.73) | 1.51(0.73to2.61) | 1.5(0.62to2.84) | 2.4(1.15to4.13) | 1.26 (0.76 to 1.77) |
| East Asia | 8.69(2.12to19.49) | 74.78(31.26to134.07) | 0.89(0.22to2.01) | 3.54(1.49to6.38) | 5.58 (5.14 to 6.03) |
| Eastern Europe | 51.64(30.53to76.97) | 96.62(58.1to139.09) | 18.09(10.68to27) | 28.78(17.25to41.54) | 1.36 (1.14 to 1.58) |
| Eastern Sub-Saharan Africa | 0.95(0.33to2) | 6.36(3.39to10.38) | 1.12(0.38to2.41) | 3.43(1.81to5.62) | 4.33 (4.09 to 4.58) |
| High-income Asia Pacific | 6.3(2.1to12.45) | 15.42(5.84to28.56) | 3.05(1.01to6.04) | 3.92(1.51to7.2) | 0.69 (0.47 to 0.92) |
| High-income North America | 63.27(35.89to93.9) | 132.43(81.09to184.22) | 19.35(11.02to28.58) | 22.55(13.86to31.32) | 0.3 (0.16 to 0.45) |
| North Africa and Middle East | 8.05(4.36to12.89) | 43.45(26.28to63.36) | 4.18(2.25to6.71) | 8.92(5.39to13.05) | 2.59 (2.52 to 2.67) |
| Oceania | 0.07(0.04to0.12) | 0.21(0.11to0.35) | 1.97(0.95to3.35) | 2.44(1.25to3.98) | 0.37 (0.04 to 0.71) |
| South Asia | 3.34(1.16to7.06) | 32.7(17.52to52.44) | 0.52(0.18to1.11) | 2.15(1.16to3.45) | 5.08 (4.89 to 5.26) |
| Southeast Asia | 3.31(1.27to6.52) | 25.38(13to42.45) | 1.13(0.43to2.23) | 3.75(1.91to6.28) | 3.85 (3.61 to 4.09) |
| Southern Latin America | 10.71(5.29to17.45) | 20.96(11.51to32.01) | 22.74(11.22to37.1) | 25.9(14.21to39.47) | 0.46 (0.26 to 0.66) |
| Southern Sub-Saharan Africa | 1.44(0.9to2.11) | 4.57(2.97to6.35) | 4.71(2.97to6.89) | 7.53(4.9to10.49) | 1.77 (1.58 to 1.96) |
| Tropical Latin America | 5.54(2.87to8.91) | 27.48(16.86to39.44) | 5.5(2.86to8.88) | 11.03(6.76to15.85) | 2.61 (2.42 to 2.8) |
| Western Europe | 78.99(42.72to123.78) | 135.2(76.11to205.8) | 14.49(7.82to22.69) | 16.56(9.31to25.19) | 0.25 (0.15 to 0.35) |
| Western Sub-Saharan Africa | 1.21(0.55to2.25) | 6.97(3.8to10.98) | 1.23(0.55to2.28) | 3.13(1.71to4.91) | 3.14 (3.04 to 3.23) |

ASDR, age-standard DALYs rate; DALYs: disability-adjusted life years; EAPC: estimated annual percentage change.
