# Supplementary material for: What was the global burden of kidney cancer attributable to high body mass index from 1990 to 2019? There existed some points noteworthy
Source: Front Nutr. 2024 Jun 5;11:1358017. doi: 10.3389/fnut.2024.1358017 (PMC11188334; doi:10.3389/fnut.2024.1358017)
Supplement: Supplementary file 5 [file Table_3.docx]

Supplementary Table 3. Death number and age-standardized morality of kidney cancer attributable to smoking for both sexes combined in 1990 and 2019, and EAPC of ASMR from 1990 to 2019 in 204 countries and territories

| Location | Death number in 1990 | Death number in 2019 | ASMR in 1990 | ASMR in 2019 | EAPC 1990-2019 |
| --- | --- | --- | --- | --- | --- |
| Afghanistan | 5.28(1.91to11.51) | 18.95(9.49to33.05) | 0.07(0.03to0.16) | 0.15(0.07to0.27) | 3.5 (2.63 to 4.38) |
| Albania | 4.49(2.24to7.23) | 20.98(10.82to34.72) | 0.23(0.11to0.37) | 0.49(0.25to0.81) | 3.58 (3.19 to 3.97) |
| Algeria | 13.47(7.23to21.92) | 71.69(40.54to109.03) | 0.11(0.06to0.18) | 0.22(0.12to0.33) | 2.25 (2.12 to 2.38) |
| American Samoa | 0.05(0.03to0.08) | 0.12(0.07to0.17) | 0.22(0.12to0.33) | 0.24(0.14to0.36) | 0.09 (-0.34 to 0.52) |
| Andorra | 0.33(0.16to0.59) | 1.07(0.54to1.73) | 0.62(0.29to1.1) | 0.75(0.38to1.21) | 0.65 (0.61 to 0.69) |
| Angola | 1.03(0.19to2.81) | 12.55(5.53to23.54) | 0.03(0to0.07) | 0.11(0.05to0.2) | 5.35 (5 to 5.7) |
| Antigua and Barbuda | 0.16(0.08to0.27) | 0.38(0.21to0.58) | 0.31(0.15to0.53) | 0.37(0.2to0.57) | 0.48 (0 to 0.97) |
| Argentina | 279.89(136.13to458.37) | 556.1(296.9to854.55) | 0.86(0.42to1.41) | 1.04(0.56to1.59) | 0.61 (0.39 to 0.83) |
| Armenia | 2.95(1.54to4.76) | 25.92(15.62to38.52) | 0.11(0.06to0.18) | 0.63(0.38to0.94) | 7.74 (6.76 to 8.73) |
| Australia | 143.72(79.52to219) | 359.84(214.04to522.79) | 0.73(0.41to1.12) | 0.86(0.51to1.24) | 0.28 (0.14 to 0.42) |
| Austria | 96.19(51.01to150.93) | 123.91(65.9to190.38) | 0.81(0.43to1.26) | 0.67(0.36to1.01) | -0.74 (-0.8 to -0.67) |
| Azerbaijan | 26.02(13.29to43.55) | 85.83(46.85to133.74) | 0.48(0.25to0.8) | 0.83(0.45to1.29) | 1.87 (1.39 to 2.34) |
| Bahamas | 0.91(0.53to1.38) | 1.95(1.11to3.03) | 0.56(0.32to0.85) | 0.48(0.27to0.74) | -0.21 (-0.49 to 0.07) |
| Bahrain | 0.87(0.49to1.34) | 5.41(3.08to8.15) | 0.51(0.28to0.79) | 0.59(0.34to0.9) | -0.08 (-0.52 to 0.37) |
| Bangladesh | 4.56(0.71to13.1) | 51.62(20.11to102.69) | 0.01(0to0.03) | 0.04(0.01to0.08) | 5.29 (5.09 to 5.5) |
| Barbados | 2.02(1.07to3.09) | 3.05(1.71to4.71) | 0.73(0.39to1.11) | 0.63(0.36to0.98) | -0.58 (-0.96 to -0.19) |
| Belarus | 30.17(16.43to47.54) | 172.82(96.08to272.73) | 0.23(0.13to0.36) | 1.08(0.6to1.71) | 5.09 (4.13 to 6.06) |
| Belgium | 90.37(47.57to142.66) | 161.83(89.23to252.08) | 0.58(0.31to0.93) | 0.68(0.37to1.06) | 0.54 (0.28 to 0.8) |
| Belize | 0.3(0.14to0.49) | 1.34(0.79to1.98) | 0.31(0.15to0.51) | 0.45(0.27to0.67) | 1.15 (1.01 to 1.3) |
| Benin | 1.06(0.44to1.94) | 7.82(3.97to12.77) | 0.05(0.02to0.1) | 0.15(0.08to0.25) | 3.74 (3.45 to 4.04) |
| Bermuda | 0.68(0.4to0.99) | 0.81(0.46to1.23) | 1.09(0.63to1.6) | 0.64(0.37to0.97) | -1.76 (-2.28 to -1.23) |
| Bhutan | 0.07(0.02to0.19) | 0.66(0.24to1.25) | 0.03(0.01to0.07) | 0.12(0.04to0.22) | 5.3 (5.06 to 5.54) |
| Bolivia (Plurinational State of) | 6.15(2.89to10.58) | 43.78(24.22to69.93) | 0.19(0.09to0.33) | 0.5(0.27to0.8) | 3.41 (3.28 to 3.53) |
| Bosnia and Herzegovina | 14.15(7.61to22.06) | 50.27(28.29to80.34) | 0.35(0.19to0.54) | 0.84(0.47to1.35) | 3.54 (3.18 to 3.9) |
| Botswana | 0.49(0.21to0.93) | 4.4(2.53to6.93) | 0.09(0.04to0.16) | 0.32(0.19to0.5) | 4.21 (3.81 to 4.61) |
| Brazil | 189.4(97.8to305.1) | 1053.2(644.45to1521.72) | 0.21(0.11to0.34) | 0.45(0.27to0.65) | 2.81 (2.61 to 3.01) |
| Brunei Darussalam | 0.17(0.05to0.38) | 1.28(0.6to2.14) | 0.2(0.05to0.43) | 0.48(0.22to0.82) | 3.99 (3.71 to 4.27) |
| Bulgaria | 32.3(18.6to47.05) | 100.15(55.89to157.41) | 0.26(0.15to0.37) | 0.74(0.41to1.16) | 6.03 (5.11 to 6.95) |
| Burkina Faso | 1.1(0.31to2.5) | 8.33(3.83to14.73) | 0.03(0.01to0.06) | 0.09(0.04to0.16) | 4.42 (4.35 to 4.48) |
| Burundi | 0.8(0.23to1.79) | 2.58(0.91to5.32) | 0.03(0.01to0.08) | 0.06(0.02to0.12) | 1.67 (1.57 to 1.78) |
| Cabo Verde | 0.11(0.04to0.19) | 1.68(0.92to2.68) | 0.05(0.02to0.08) | 0.37(0.2to0.6) | 6.9 (6.32 to 7.48) |
| Cambodia | 0.99(0.21to2.57) | 9.62(4.07to17.71) | 0.02(0to0.06) | 0.08(0.03to0.15) | 5.03 (4.78 to 5.29) |
| Cameroon | 4.27(2.2to6.99) | 18.89(9.78to30.22) | 0.09(0.05to0.16) | 0.16(0.08to0.25) | 1.64 (1.53 to 1.74) |
| Canada | 155.54(84.67to240.96) | 534.21(315.03to788.81) | 0.49(0.27to0.75) | 0.77(0.45to1.13) | 1.9 (1.53 to 2.27) |
| Central African Republic | 0.37(0.1to0.83) | 1.05(0.36to2.27) | 0.03(0.01to0.07) | 0.05(0.02to0.1) | 1.75 (1.55 to 1.94) |
| Chad | 0.48(0.13to1.08) | 2.89(1.26to5.37) | 0.02(0to0.04) | 0.05(0.02to0.09) | 3.72 (3.63 to 3.81) |
| Chile | 76.26(39.3to120.21) | 254.74(145.19to383.65) | 0.76(0.39to1.2) | 1.06(0.6to1.59) | 1.43 (1.3 to 1.55) |
| China | 275.97(64.58to626.64) | 2589.07(1081.71to4699.44) | 0.03(0.01to0.08) | 0.13(0.05to0.24) | 5.61 (5.13 to 6.09) |
| Colombia | 27.54(13.87to45.13) | 147.67(77.64to243.37) | 0.16(0.08to0.26) | 0.28(0.15to0.46) | 1.98 (1.85 to 2.1) |
| Comoros | 0.14(0.05to0.29) | 0.71(0.34to1.25) | 0.06(0.02to0.13) | 0.15(0.07to0.26) | 2.85 (2.74 to 2.97) |
| Congo | 1.24(0.51to2.33) | 6.27(3.14to10.93) | 0.11(0.05to0.22) | 0.23(0.12to0.4) | 2.41 (2.25 to 2.57) |
| Cook Islands | 0.02(0.01to0.03) | 0.05(0.03to0.07) | 0.14(0.07to0.22) | 0.19(0.11to0.29) | 0.83 (0.63 to 1.03) |
| Costa Rica | 4.08(2.15to6.5) | 24.38(12.66to39.86) | 0.24(0.12to0.37) | 0.47(0.24to0.77) | 2.61 (2.44 to 2.78) |
| Croatia | 19.58(10.63to29.87) | 102.45(58.23to156.97) | 0.3(0.16to0.47) | 1.15(0.65to1.76) | 4.97 (4.32 to 5.61) |
| Cuba | 45.32(26.16to68.58) | 76.99(43.02to122.39) | 0.44(0.26to0.67) | 0.41(0.23to0.66) | -0.01 (-0.65 to 0.64) |
| Cyprus | 1.08(0.51to1.86) | 6.22(3.31to9.88) | 0.14(0.06to0.24) | 0.32(0.17to0.51) | 3.59 (3.22 to 3.97) |
| Czechia | 153.26(86.54to228.39) | 383.53(225.07to570.77) | 1.12(0.63to1.67) | 1.79(1.05to2.67) | 1.39 (0.88 to 1.9) |
| C么te d'Ivoire | 3.1(1.38to5.74) | 16.1(8.05to27.95) | 0.07(0.03to0.14) | 0.14(0.07to0.24) | 1.95 (1.85 to 2.06) |
| Democratic People's Republic of Korea | 5.45(0.97to13.89) | 13.59(2.69to33.68) | 0.03(0.01to0.09) | 0.04(0.01to0.11) | 0.8 (0.75 to 0.85) |
| Democratic Republic of the Congo | 9.85(4.03to18.5) | 23.73(9.65to45.53) | 0.06(0.03to0.12) | 0.07(0.03to0.13) | -0.68 (-1.39 to 0.03) |
| Denmark | 33.59(17.6to53.66) | 85.89(47.38to134.78) | 0.42(0.22to0.66) | 0.75(0.42to1.17) | 1.33 (0.73 to 1.93) |
| Djibouti | 0.05(0.01to0.11) | 1(0.44to1.87) | 0.03(0.01to0.08) | 0.17(0.07to0.31) | 6.54 (6.23 to 6.85) |
| Dominica | 0.35(0.19to0.55) | 0.51(0.29to0.8) | 0.52(0.28to0.79) | 0.57(0.32to0.9) | 0.55 (0.21 to 0.88) |
| Dominican Republic | 6.69(2.92to11.81) | 44.71(22.25to78.23) | 0.17(0.07to0.3) | 0.47(0.23to0.82) | 4.28 (3.57 to 5) |
| Ecuador | 15.52(9.2to23.02) | 78.28(44.91to118.74) | 0.28(0.16to0.42) | 0.52(0.3to0.79) | 2.63 (2.22 to 3.03) |
| Egypt | 34.04(19.41to51.43) | 186.74(101.28to318.99) | 0.11(0.06to0.18) | 0.28(0.15to0.49) | 2.94 (2.73 to 3.16) |
| El Salvador | 4.41(2.2to7.21) | 19.56(10.14to31.98) | 0.15(0.07to0.24) | 0.33(0.17to0.54) | 2.74 (2.47 to 3.01) |
| Equatorial Guinea | 0.05(0.01to0.14) | 1.71(0.78to3.38) | 0.03(0.01to0.07) | 0.36(0.17to0.69) | 11.48 (10.6 to 12.37) |
| Eritrea | 0.23(0.07to0.52) | 2.75(1.19to5.03) | 0.02(0.01to0.05) | 0.11(0.05to0.2) | 5.34 (4.75 to 5.94) |
| Estonia | 8.26(4.69to12.61) | 42.06(24.58to63.59) | 0.4(0.23to0.61) | 1.55(0.91to2.35) | 5.02 (4.23 to 5.8) |
| Eswatini | 0.6(0.32to0.98) | 2.54(1.31to4.28) | 0.21(0.11to0.34) | 0.45(0.24to0.74) | 2.32 (1.52 to 3.13) |
| Ethiopia | 7.08(1.31to20.61) | 42.08(15.48to83.32) | 0.04(0.01to0.11) | 0.1(0.04to0.21) | 3.86 (3.27 to 4.45) |
| Fiji | 0.44(0.23to0.71) | 1.37(0.78to2.1) | 0.12(0.06to0.19) | 0.19(0.11to0.29) | 1.18 (0.91 to 1.45) |
| Finland | 51.01(27.39to80.6) | 102.27(56.59to155.62) | 0.72(0.38to1.13) | 0.81(0.45to1.22) | 0.24 (0.11 to 0.37) |
| France | 486.54(256.86to771.41) | 1010.41(562.99to1571.17) | 0.59(0.31to0.94) | 0.71(0.4to1.1) | 0.62 (0.55 to 0.69) |
| Gabon | 0.71(0.28to1.52) | 4.44(2.22to7.88) | 0.12(0.05to0.27) | 0.41(0.21to0.73) | 3.99 (3.68 to 4.29) |
| Gambia | 0.14(0.06to0.27) | 1.19(0.59to2.06) | 0.04(0.02to0.08) | 0.12(0.06to0.21) | 3.77 (3.58 to 3.95) |
| Georgia | 24.23(13.04to39.55) | 36.21(20.14to54.82) | 0.38(0.21to0.62) | 0.63(0.35to0.95) | 2.37 (1.82 to 2.92) |
| Germany | 897.6(493.45to1399.97) | 1738.1(951.39to2652.94) | 0.72(0.4to1.13) | 0.87(0.48to1.33) | 0.36 (0.23 to 0.49) |
| Ghana | 4.32(1.74to8.14) | 31.07(17.67to50.06) | 0.06(0.02to0.12) | 0.19(0.11to0.3) | 3.11 (2.68 to 3.55) |
| Greece | 75.55(38.93to119.39) | 155.29(81.97to240.25) | 0.49(0.25to0.78) | 0.64(0.34to0.99) | 0.48 (0.21 to 0.75) |
| Greenland | 0.26(0.14to0.4) | 1.02(0.56to1.57) | 0.76(0.41to1.19) | 1.48(0.81to2.28) | 2 (1.61 to 2.39) |
| Grenada | 0.16(0.08to0.27) | 0.43(0.25to0.66) | 0.24(0.12to0.4) | 0.38(0.22to0.58) | 1.3 (0.8 to 1.81) |
| Guam | 0.26(0.13to0.41) | 0.59(0.32to0.93) | 0.35(0.16to0.56) | 0.31(0.17to0.49) | -0.27 (-0.61 to 0.06) |
| Guatemala | 3.89(1.41to7.29) | 28.65(14.5to48.58) | 0.1(0.04to0.19) | 0.26(0.13to0.44) | 3.57 (3.02 to 4.12) |
| Guinea | 1.29(0.53to2.48) | 4.45(2.15to7.65) | 0.04(0.02to0.07) | 0.08(0.04to0.14) | 2.49 (2.44 to 2.53) |
| Guinea-Bissau | 0.18(0.06to0.39) | 0.73(0.33to1.3) | 0.04(0.01to0.1) | 0.09(0.04to0.17) | 2.5 (2.36 to 2.64) |
| Guyana | 1.39(0.68to2.39) | 2.72(1.44to4.46) | 0.34(0.17to0.59) | 0.4(0.22to0.66) | 0.68 (0.4 to 0.95) |
| Haiti | 3.15(1.12to6.32) | 7.6(3.04to15.35) | 0.09(0.03to0.19) | 0.1(0.04to0.21) | 0.68 (0.38 to 0.97) |
| Honduras | 2.12(0.87to3.8) | 19.06(9.49to35.42) | 0.1(0.04to0.19) | 0.33(0.16to0.61) | 4.21 (3.88 to 4.54) |
| Hungary | 165.78(101.83to241.35) | 254.86(158.07to380.37) | 1.13(0.69to1.65) | 1.33(0.83to1.99) | 0.38 (0.04 to 0.73) |
| Iceland | 2.87(1.51to4.42) | 6.08(3.3to9.41) | 1.01(0.53to1.55) | 1.09(0.59to1.67) | 0.26 (0.04 to 0.49) |
| India | 88.49(32.3to185.12) | 930.37(494.77to1527.72) | 0.02(0.01to0.04) | 0.08(0.04to0.13) | 4.9 (4.7 to 5.11) |
| Indonesia | 27.69(7.69to60.91) | 335.43(157.37to650.23) | 0.03(0.01to0.06) | 0.15(0.07to0.29) | 6.27 (6.16 to 6.37) |
| Iran (Islamic Republic of) | 31.06(14.8to51.84) | 196.15(121.05to284.06) | 0.13(0.06to0.21) | 0.28(0.17to0.4) | 2.85 (2.61 to 3.09) |
| Iraq | 21.01(9.8to35.46) | 103.82(57.68to165.14) | 0.27(0.12to0.46) | 0.45(0.25to0.7) | 2.07 (1.68 to 2.47) |
| Ireland | 21.43(11.16to33.62) | 59.73(32.68to91.67) | 0.53(0.28to0.83) | 0.8(0.44to1.22) | 1.47 (1.16 to 1.77) |
| Israel | 24.98(13.37to40.01) | 74.23(39.59to116.43) | 0.52(0.28to0.84) | 0.63(0.34to0.99) | 0.31 (-0.01 to 0.64) |
| Italy | 489.77(259.03to775.88) | 944.26(515.84to1484) | 0.55(0.29to0.87) | 0.63(0.35to0.98) | 0.34 (0.23 to 0.44) |
| Jamaica | 4.97(2.75to7.84) | 8.37(4.76to13.4) | 0.29(0.16to0.45) | 0.28(0.16to0.45) | 0.29 (-0.15 to 0.73) |
| Japan | 232.13(76.8to459.19) | 655.39(223.37to1275.8) | 0.14(0.05to0.27) | 0.17(0.06to0.33) | 0.56 (0.38 to 0.75) |
| Jordan | 2.3(1.3to3.52) | 25.92(15.46to37.09) | 0.18(0.1to0.27) | 0.41(0.24to0.6) | 3.53 (3.27 to 3.8) |
| Kazakhstan | 77.78(40.16to126.91) | 154.77(92.53to222.18) | 0.61(0.31to1) | 0.87(0.52to1.25) | 0.85 (0.48 to 1.22) |
| Kenya | 2.67(1to5.17) | 27.84(14.91to44.91) | 0.03(0.01to0.06) | 0.12(0.06to0.2) | 5.56 (5.23 to 5.89) |
| Kiribati | 0.13(0.06to0.23) | 0.33(0.15to0.59) | 0.32(0.14to0.56) | 0.41(0.18to0.73) | 0.24 (-0.22 to 0.71) |
| Kuwait | 1.81(1.09to2.67) | 11.33(6.78to16.67) | 0.29(0.17to0.43) | 0.45(0.26to0.66) | 1.3 (0.75 to 1.86) |
| Kyrgyzstan | 4.9(2.54to7.92) | 17.55(9.58to27.5) | 0.16(0.08to0.26) | 0.37(0.2to0.57) | 2.06 (1.61 to 2.5) |
| Lao People's Democratic Republic | 0.6(0.14to1.53) | 4.95(2.25to8.69) | 0.03(0.01to0.07) | 0.11(0.05to0.19) | 4.98 (4.87 to 5.1) |
| Latvia | 12.38(6.67to19) | 55.13(32.17to84.32) | 0.34(0.19to0.53) | 1.37(0.8to2.08) | 4.67 (3.91 to 5.44) |
| Lebanon | 5.15(2.56to8.89) | 29.98(15.71to49.11) | 0.23(0.11to0.39) | 0.58(0.31to0.95) | 3.96 (3.69 to 4.23) |
| Lesotho | 0.58(0.23to1.11) | 2.96(1.56to4.83) | 0.06(0.02to0.12) | 0.24(0.13to0.38) | 5.35 (5.05 to 5.65) |
| Liberia | 0.84(0.39to1.45) | 3.37(1.62to5.95) | 0.07(0.03to0.13) | 0.15(0.07to0.27) | 3.71 (2.85 to 4.57) |
| Libya | 5.64(2.73to9.59) | 29.17(15.33to46.03) | 0.31(0.15to0.52) | 0.59(0.31to0.92) | 2.42 (2.21 to 2.63) |
| Lithuania | 18.07(9.79to28.08) | 80.69(44.76to121.78) | 0.4(0.22to0.62) | 1.4(0.78to2.11) | 4.4 (3.63 to 5.18) |
| Luxembourg | 2.09(1.15to3.25) | 3.51(1.97to5.46) | 0.38(0.21to0.59) | 0.35(0.19to0.54) | -0.47 (-0.55 to -0.38) |
| Madagascar | 1.68(0.62to3.36) | 8.88(4.07to15.74) | 0.03(0.01to0.07) | 0.08(0.04to0.14) | 3.75 (3.45 to 4.05) |
| Malawi | 3.34(0.85to7.81) | 19.8(9.14to34.18) | 0.09(0.02to0.2) | 0.28(0.13to0.49) | 4.77 (4.47 to 5.07) |
| Malaysia | 8.17(3.63to14.71) | 70.54(38.01to116.27) | 0.09(0.04to0.16) | 0.27(0.14to0.44) | 3.72 (3.55 to 3.89) |
| Maldives | 0.03(0.01to0.07) | 0.34(0.16to0.58) | 0.03(0.01to0.08) | 0.1(0.05to0.19) | 4.4 (4.22 to 4.58) |
| Mali | 1.27(0.41to2.55) | 7.23(3.45to12.5) | 0.03(0.01to0.06) | 0.08(0.04to0.14) | 3.52 (3.4 to 3.64) |
| Malta | 1.81(0.88to2.98) | 4.87(2.5to7.81) | 0.43(0.21to0.7) | 0.52(0.27to0.84) | 0.94 (0.67 to 1.2) |
| Marshall Islands | 0.01(0to0.03) | 0.05(0.02to0.09) | 0.08(0.03to0.16) | 0.13(0.06to0.24) | 1.39 (1.14 to 1.64) |
| Mauritania | 1.11(0.55to1.91) | 4.18(2.32to6.66) | 0.11(0.06to0.2) | 0.2(0.11to0.32) | 1.98 (1.91 to 2.05) |
| Mauritius | 0.65(0.34to1.05) | 3.96(2.14to6.27) | 0.09(0.04to0.14) | 0.23(0.12to0.36) | 3.38 (3.08 to 3.68) |
| Mexico | 175.61(97.34to267.27) | 839.67(483.08to1248.31) | 0.41(0.23to0.63) | 0.71(0.41to1.06) | 1.88 (1.77 to 1.99) |
| Micronesia (Federated States of) | 0.07(0.04to0.13) | 0.17(0.08to0.29) | 0.15(0.07to0.26) | 0.21(0.1to0.37) | 0.77 (0.46 to 1.08) |
| Monaco | 0.52(0.26to0.85) | 1(0.52to1.56) | 0.74(0.38to1.21) | 1.04(0.55to1.62) | 1.31 (1.1 to 1.52) |
| Mongolia | 1.95(0.94to3.35) | 10.8(5.7to17.6) | 0.19(0.09to0.33) | 0.48(0.25to0.78) | 3.47 (3.3 to 3.63) |
| Montenegro | 3.65(2.13to5.4) | 9.06(5.39to13.5) | 0.59(0.34to0.87) | 0.92(0.54to1.37) | 1.96 (1.82 to 2.1) |
| Morocco | 9.56(4.77to15.96) | 58.48(29.99to93.19) | 0.07(0.03to0.11) | 0.19(0.1to0.3) | 3.41 (3.21 to 3.62) |
| Mozambique | 1.11(0.25to2.67) | 9.82(4.08to18.65) | 0.02(0to0.05) | 0.09(0.04to0.17) | 6.3 (6.03 to 6.57) |
| Myanmar | 5.35(1.03to14.26) | 48.06(22.18to83.59) | 0.02(0to0.06) | 0.1(0.05to0.18) | 6.02 (5.71 to 6.33) |
| Namibia | 0.95(0.5to1.55) | 4.46(2.57to7.04) | 0.13(0.07to0.22) | 0.33(0.19to0.51) | 3.09 (2.9 to 3.28) |
| Nauru | 0.01(0.01to0.02) | 0.01(0.01to0.02) | 0.25(0.12to0.46) | 0.27(0.13to0.45) | -0.43 (-0.76 to -0.1) |
| Nepal | 1.09(0.23to2.92) | 15.93(5.78to32.94) | 0.01(0to0.03) | 0.07(0.03to0.15) | 7.04 (6.81 to 7.27) |
| Netherlands | 122.42(62.86to194.03) | 280.15(147.52to441.34) | 0.62(0.32to0.98) | 0.81(0.42to1.28) | 0.84 (0.68 to 1) |
| New Zealand | 23.93(13.14to37.1) | 61.36(35.14to90.85) | 0.61(0.34to0.95) | 0.79(0.46to1.16) | 1.02 (0.89 to 1.15) |
| Nicaragua | 1.75(0.79to3.09) | 14.4(7.51to23.25) | 0.11(0.05to0.2) | 0.34(0.18to0.54) | 3.81 (3.32 to 4.31) |
| Niger | 0.78(0.29to1.54) | 3.88(1.74to6.97) | 0.03(0.01to0.06) | 0.05(0.02to0.09) | 1.95 (1.85 to 2.05) |
| Nigeria | 17.81(6.98to35.48) | 101.35(50.25to170.01) | 0.04(0.02to0.08) | 0.11(0.06to0.19) | 3.75 (3.67 to 3.84) |
| Niue | 0(0to0.01) | 0.01(0to0.01) | 0.18(0.09to0.31) | 0.29(0.15to0.47) | 1.39 (1.13 to 1.65) |
| North Macedonia | 3.02(1.69to4.69) | 17.49(9.97to27.67) | 0.16(0.09to0.25) | 0.55(0.31to0.85) | 5.31 (4.74 to 5.89) |
| Northern Mariana Islands | 0.09(0.05to0.14) | 0.21(0.12to0.31) | 0.41(0.22to0.67) | 0.4(0.22to0.59) | -0.56 (-1.14 to 0.03) |
| Norway | 35.85(18.55to56.91) | 66.06(35.14to103.63) | 0.53(0.27to0.83) | 0.68(0.36to1.06) | 1 (0.81 to 1.19) |
| Oman | 0.59(0.26to1.05) | 6.26(3.74to9.31) | 0.08(0.04to0.15) | 0.38(0.22to0.55) | 5.74 (5.2 to 6.28) |
| Pakistan | 20.37(5.17to46.28) | 156.8(73.97to273.8) | 0.04(0.01to0.08) | 0.14(0.07to0.25) | 5.31 (4.88 to 5.74) |
| Palau | 0.01(0.01to0.02) | 0.04(0.02to0.06) | 0.11(0.06to0.19) | 0.17(0.09to0.27) | 1.11 (0.81 to 1.4) |
| Palestine | 1.42(0.59to2.65) | 7.21(3.94to11.19) | 0.16(0.07to0.3) | 0.3(0.16to0.48) | 1.85 (1.54 to 2.16) |
| Panama | 1.66(0.57to3.17) | 15.68(8.21to26.09) | 0.11(0.04to0.21) | 0.38(0.2to0.63) | 5.05 (4.85 to 5.25) |
| Papua New Guinea | 0.56(0.17to1.27) | 2.2(0.79to4.58) | 0.03(0.01to0.07) | 0.04(0.01to0.09) | 0.81 (0.58 to 1.04) |
| Paraguay | 5.13(2.64to8.38) | 19.83(10.15to33.36) | 0.23(0.12to0.37) | 0.35(0.18to0.59) | 1.41 (1.29 to 1.52) |
| Peru | 36.76(17.7to62.32) | 146.88(75.17to244.66) | 0.3(0.15to0.51) | 0.46(0.23to0.77) | 1.4 (1.11 to 1.68) |
| Philippines | 20.86(8.76to38.03) | 119.33(59.71to196.81) | 0.07(0.03to0.12) | 0.15(0.07to0.24) | 2.38 (2.1 to 2.66) |
| Poland | 162.41(90.85to243.52) | 1015.93(585.22to1488.16) | 0.37(0.21to0.55) | 1.44(0.83to2.12) | 4.81 (3.75 to 5.89) |
| Portugal | 38.78(18.53to63.47) | 94.19(48.47to150.79) | 0.28(0.13to0.46) | 0.39(0.2to0.62) | 0.92 (0.69 to 1.15) |
| Puerto Rico | 18.37(10.45to27.31) | 35.35(20.44to53.93) | 0.51(0.29to0.76) | 0.52(0.3to0.8) | 0.47 (0.2 to 0.75) |
| Qatar | 0.65(0.35to1.05) | 7.56(4.16to11.94) | 0.66(0.34to1.08) | 1.29(0.69to2.01) | 2.25 (1.95 to 2.54) |
| Republic of Korea | 21.91(6.99to44.11) | 173.75(71.26to310.08) | 0.08(0.02to0.15) | 0.2(0.08to0.35) | 3 (2.35 to 3.67) |
| Republic of Moldova | 13.95(7.74to21.6) | 37.95(22.65to55.95) | 0.3(0.17to0.47) | 0.65(0.39to0.95) | 3.41 (2.89 to 3.95) |
| Romania | 104.16(64.3to150.12) | 291.35(183.04to422.83) | 0.37(0.22to0.53) | 0.82(0.51to1.2) | 2.94 (2.83 to 3.05) |
| Russian Federation | 1327.42(794.19to1959.91) | 2720.66(1633.28to3940.26) | 0.72(0.43to1.07) | 1.15(0.69to1.66) | 1.41 (1.19 to 1.62) |
| Rwanda | 1.41(0.37to3.12) | 7.71(3.4to13.49) | 0.05(0.01to0.11) | 0.13(0.06to0.23) | 3.7 (3.18 to 4.21) |
| Saint Kitts and Nevis | 0.33(0.19to0.52) | 0.43(0.24to0.66) | 0.92(0.52to1.43) | 0.63(0.36to0.96) | -1.32 (-1.89 to -0.74) |
| Saint Lucia | 0.3(0.15to0.48) | 0.7(0.39to1.1) | 0.34(0.17to0.56) | 0.32(0.18to0.51) | -0.61 (-1.05 to -0.16) |
| Saint Vincent and the Grenadines | 0.24(0.12to0.41) | 0.52(0.3to0.79) | 0.34(0.16to0.57) | 0.38(0.22to0.58) | 0.74 (0.23 to 1.25) |
| Samoa | 0.17(0.09to0.26) | 0.3(0.16to0.49) | 0.19(0.1to0.31) | 0.21(0.11to0.34) | -0.12 (-0.31 to 0.06) |
| San Marino | 0.13(0.07to0.21) | 0.33(0.16to0.57) | 0.39(0.2to0.62) | 0.5(0.24to0.88) | 1.15 (1.03 to 1.27) |
| Sao Tome and Principe | 0.03(0.01to0.06) | 0.19(0.09to0.34) | 0.05(0.02to0.1) | 0.17(0.09to0.32) | 4.22 (4.14 to 4.29) |
| Saudi Arabia | 7.68(3.77to13.03) | 89.69(51.47to138.08) | 0.13(0.06to0.22) | 0.47(0.28to0.71) | 4.12 (3.75 to 4.49) |
| Senegal | 1.93(0.86to3.44) | 9.61(5to15.63) | 0.06(0.03to0.11) | 0.13(0.06to0.2) | 2.68 (2.52 to 2.85) |
| Serbia | 67.4(36.9to103.11) | 165.66(94.1to253.08) | 0.6(0.32to0.92) | 1.04(0.59to1.59) | 2.16 (2.04 to 2.28) |
| Seychelles | 0.08(0.04to0.13) | 0.33(0.18to0.53) | 0.14(0.07to0.23) | 0.29(0.15to0.46) | 1.54 (1.01 to 2.06) |
| Sierra Leone | 0.59(0.17to1.32) | 2.74(1.2to5.05) | 0.03(0.01to0.07) | 0.07(0.03to0.13) | 3 (2.7 to 3.29) |
| Singapore | 3.04(0.93to6.01) | 20.91(10.52to34.28) | 0.14(0.04to0.28) | 0.27(0.13to0.44) | 2.21 (1.89 to 2.53) |
| Slovakia | 39.33(22.87to58.37) | 127.32(74.12to192.86) | 0.65(0.38to0.97) | 1.37(0.8to2.07) | 2.41 (1.98 to 2.84) |
| Slovenia | 13.3(7.36to20.42) | 46.31(26.34to72.81) | 0.54(0.3to0.84) | 1.06(0.59to1.67) | 2.57 (2.18 to 2.95) |
| Solomon Islands | 0.1(0.03to0.22) | 0.42(0.18to0.81) | 0.07(0.02to0.15) | 0.12(0.05to0.22) | 1.39 (0.9 to 1.88) |
| Somalia | 0.55(0.1to1.61) | 2(0.32to5.65) | 0.02(0to0.06) | 0.03(0to0.09) | 1.63 (1.48 to 1.77) |
| South Africa | 44.41(27.86to64.97) | 145.97(96.15to202.39) | 0.21(0.13to0.31) | 0.34(0.22to0.47) | 1.79 (1.61 to 1.96) |
| South Sudan | 1.79(0.58to4.71) | 6.01(2.67to12.61) | 0.08(0.02to0.2) | 0.16(0.07to0.34) | 3 (2.84 to 3.15) |
| Spain | 234.73(126.68to367.64) | 632.09(352.37to978.51) | 0.43(0.23to0.67) | 0.65(0.36to1.01) | 1.27 (1.12 to 1.43) |
| Sri Lanka | 23.15(10.23to40.59) | 84.4(41.46to145.24) | 0.22(0.09to0.38) | 0.33(0.16to0.57) | -0.24 (-1.14 to 0.66) |
| Sudan | 4.83(1.72to10.05) | 41.42(17.45to83.6) | 0.05(0.02to0.11) | 0.22(0.09to0.45) | 5.59 (5.18 to 5.99) |
| Suriname | 0.83(0.43to1.31) | 2.15(1.17to3.44) | 0.31(0.16to0.49) | 0.35(0.19to0.56) | 0.7 (0.18 to 1.22) |
| Sweden | 128.35(65.72to205.81) | 171.42(94.23to267.83) | 0.86(0.44to1.38) | 0.79(0.44to1.23) | -0.5 (-0.61 to -0.39) |
| Switzerland | 30.72(16.19to48.61) | 88.54(47.94to137.76) | 0.29(0.15to0.46) | 0.49(0.26to0.75) | 1.08 (0.57 to 1.6) |
| Syrian Arab Republic | 4.2(2.13to6.97) | 18.67(10.05to29.97) | 0.08(0.04to0.13) | 0.15(0.08to0.24) | 2.17 (1.95 to 2.39) |
| Taiwan (Province of China) | 15(6.36to26.49) | 167.14(75.59to290.06) | 0.1(0.04to0.17) | 0.42(0.19to0.73) | 4.82 (4.11 to 5.53) |
| Tajikistan | 3.98(1.46to8.12) | 12.58(5.57to22.3) | 0.14(0.05to0.29) | 0.23(0.1to0.42) | 1.74 (1.11 to 2.37) |
| Thailand | 14.76(4.97to29.61) | 122.23(58.24to209.43) | 0.04(0.01to0.08) | 0.12(0.06to0.2) | 3.08 (2.64 to 3.52) |
| Timor-Leste | 0.05(0.01to0.13) | 0.37(0.11to0.84) | 0.02(0to0.04) | 0.04(0.01to0.1) | 4.36 (3.72 to 5.01) |
| Togo | 0.56(0.22to1.07) | 4.12(2.11to6.9) | 0.04(0.02to0.09) | 0.11(0.05to0.18) | 2.79 (2.66 to 2.91) |
| Tokelau | 0(0to0) | 0(0to0) | 0.09(0.04to0.17) | 0.17(0.09to0.29) | 1.96 (1.8 to 2.12) |
| Tonga | 0.07(0.03to0.11) | 0.15(0.08to0.25) | 0.11(0.06to0.2) | 0.19(0.1to0.31) | 1.1 (0.49 to 1.72) |
| Trinidad and Tobago | 5.64(3.34to8.36) | 7.95(4.48to12.63) | 0.65(0.39to0.97) | 0.43(0.24to0.69) | -1.83 (-2.47 to -1.18) |
| Tunisia | 5.95(3to10.05) | 33.75(17.58to56.52) | 0.12(0.06to0.2) | 0.27(0.14to0.45) | 2.87 (2.81 to 2.93) |
| Turkey | 113.77(60.37to184.52) | 459.6(257.03to694.87) | 0.31(0.16to0.51) | 0.52(0.29to0.79) | 1.66 (1.56 to 1.75) |
| Turkmenistan | 5.03(2.58to8.13) | 37.16(20.53to56.61) | 0.25(0.13to0.41) | 0.86(0.48to1.31) | 5.15 (4.72 to 5.59) |
| Tuvalu | 0.01(0to0.01) | 0.02(0.01to0.03) | 0.09(0.03to0.17) | 0.15(0.07to0.28) | 1.26 (0.93 to 1.59) |
| Uganda | 1.85(0.48to4.22) | 24.11(11.33to41.44) | 0.03(0.01to0.07) | 0.17(0.08to0.3) | 7.2 (6.91 to 7.49) |
| Ukraine | 490.78(277.19to762.97) | 786.46(475.7to1199.12) | 0.67(0.38to1.05) | 1.05(0.63to1.62) | 1.4 (1.16 to 1.63) |
| United Arab Emirates | 3.3(1.09to8.73) | 81.28(27.35to149.82) | 0.61(0.17to1.66) | 1.5(0.44to2.83) | 3.2 (2.86 to 3.55) |
| United Kingdom | 585.35(313.24to908.94) | 1163.57(660.73to1732.87) | 0.66(0.35to1.02) | 0.92(0.52to1.36) | 1.05 (0.92 to 1.17) |
| United Republic of Tanzania | 7.63(3.15to14.44) | 48.98(25.62to82.88) | 0.07(0.03to0.13) | 0.2(0.1to0.34) | 3.98 (3.7 to 4.26) |
| United States of America | 2370.89(1326.48to3556.08) | 5312.18(3222.76to7476.08) | 0.76(0.43to1.13) | 0.95(0.58to1.33) | 0.55 (0.4 to 0.69) |
| United States Virgin Islands | 0.56(0.32to0.85) | 1.88(1.09to2.82) | 0.64(0.36to0.98) | 1.03(0.6to1.55) | 1.94 (1.62 to 2.27) |
| Uruguay | 37.95(19.06to60.96) | 69.37(36.98to106.5) | 0.99(0.5to1.58) | 1.33(0.72to2.04) | 0.83 (0.71 to 0.94) |
| Uzbekistan | 15.83(6.72to29.1) | 92.36(52.23to143.56) | 0.13(0.06to0.25) | 0.4(0.22to0.61) | 4.01 (3.89 to 4.13) |
| Vanuatu | 0.05(0.02to0.1) | 0.21(0.09to0.39) | 0.07(0.03to0.15) | 0.11(0.05to0.22) | 1.46 (1.32 to 1.6) |
| Venezuela (Bolivarian Republic of) | 47.7(25.62to74.62) | 154.58(79.02to260.03) | 0.47(0.25to0.73) | 0.52(0.27to0.87) | 0.58 (0.09 to 1.07) |
| Viet Nam | 5.55(1.16to13.96) | 61.8(24.45to117.37) | 0.01(0to0.04) | 0.07(0.03to0.12) | 6.29 (5.98 to 6.59) |
| Yemen | 1.63(0.45to3.7) | 11.1(5.08to19.65) | 0.03(0.01to0.07) | 0.08(0.04to0.15) | 4.12 (3.83 to 4.41) |
| Zambia | 2.19(0.73to4.51) | 14.97(7.02to26.87) | 0.08(0.02to0.16) | 0.22(0.1to0.4) | 3.71 (3.45 to 3.97) |
| Zimbabwe | 2.95(1.54to4.86) | 9.55(4.94to15.12) | 0.07(0.04to0.12) | 0.13(0.07to0.22) | 2.02 (1.59 to 2.44) |

ASMR, age-standard morality rate; EAPC, estimated annual percentage change.
