# Supplementary material for: What was the global burden of kidney cancer attributable to high body mass index from 1990 to 2019? There existed some points noteworthy
Source: Front Nutr. 2024 Jun 5;11:1358017. doi: 10.3389/fnut.2024.1358017 (PMC11188334; doi:10.3389/fnut.2024.1358017)
Supplement: Supplementary file 6 [file Table_4.docx]

Supplementary Table 4. DALYs number and age-standardized DALYs rate of kidney cancer attributable to smoking for both sexes combined in 1990 and 2019, and EAPC of ASMR from 1990 to 2019 in 204 countries and territories

| Location | DALYs number in 1990 | DALYs number in 2019 | ASDR in 1990 | ASDR in 2019 | EAPC 1990-2019 |
| --- | --- | --- | --- | --- | --- |
| Afghanistan | 150.94(56.32to323.9) | 584.65(289.61to1044.97) | 1.95(0.72to4.19) | 3.93(1.99to6.78) | 3.3 (2.44 to 4.16) |
| Albania | 112.97(56.3to183.23) | 477.74(247.24to791.45) | 5.22(2.6to8.45) | 11.38(5.89to18.63) | 3.65 (3.25 to 4.06) |
| Algeria | 393.03(210.61to641.53) | 2080.71(1170.19to3152.14) | 2.91(1.56to4.72) | 5.58(3.15to8.49) | 2.24 (2.12 to 2.36) |
| American Samoa | 1.61(0.98to2.43) | 3.45(2.14to5.07) | 5.86(3.55to8.81) | 6.65(4.11to9.81) | 0.11 (-0.33 to 0.55) |
| Andorra | 8.28(3.9to14.81) | 23.99(12.09to39.32) | 14.39(6.81to25.55) | 17.33(8.75to28.28) | 0.61 (0.57 to 0.66) |
| Angola | 32.18(6.09to86.6) | 393.09(177.67to730.02) | 0.69(0.13to1.87) | 2.86(1.26to5.37) | 5.37 (5.01 to 5.74) |
| Antigua and Barbuda | 4.36(2.14to7.23) | 10.47(5.97to16.06) | 8.79(4.37to14.53) | 9.72(5.54to14.87) | 0.29 (-0.19 to 0.76) |
| Argentina | 7654.13(3668.66to12568.46) | 13430.54(7186.4to20701.67) | 23.4(11.19to38.44) | 25.78(13.8to39.68) | 0.27 (-0.01 to 0.56) |
| Armenia | 84.04(44.3to134.68) | 616.11(375to914.05) | 2.86(1.49to4.59) | 14.9(9.13to22.07) | 7.29 (6.35 to 8.23) |
| Australia | 3376.33(1892.15to5114.26) | 7578.79(4550.23to10923.72) | 17.5(9.8to26.51) | 19.74(11.87to28.28) | 0.2 (0.08 to 0.32) |
| Austria | 2050.1(1085.95to3217.11) | 2335.32(1272.62to3552.23) | 18.28(9.57to28.79) | 14.05(7.81to21.42) | -1.01 (-1.09 to -0.93) |
| Azerbaijan | 812.17(415.22to1374.48) | 2647.88(1430.29to4124.92) | 14.36(7.38to24.01) | 23.14(12.56to36.08) | 1.6 (1.13 to 2.06) |
| Bahamas | 29.53(17.42to44.27) | 60.6(34.3to93.48) | 16.31(9.61to24.61) | 14.07(8.01to21.84) | -0.23 (-0.52 to 0.07) |
| Bahrain | 25.87(14.79to39.77) | 168.63(96.34to250.09) | 12.14(6.85to18.88) | 13.37(7.64to20.1) | -0.37 (-0.79 to 0.06) |
| Bangladesh | 132.13(21.36to375.71) | 1519.1(613.42to2961.11) | 0.25(0.04to0.72) | 1.08(0.43to2.1) | 5.7 (5.46 to 5.94) |
| Barbados | 51.35(27.68to78.6) | 78.24(43.84to120.94) | 19.88(10.77to30.29) | 17.15(9.63to26.41) | -0.62 (-1.01 to -0.22) |
| Belarus | 818.43(447.2to1282.98) | 4366.99(2386.3to6907.05) | 6.24(3.4to9.81) | 28.15(15.34to44.63) | 4.88 (3.9 to 5.86) |
| Belgium | 1960.25(1037.57to3123.24) | 3056.87(1677.1to4774.82) | 13.29(7.04to21.21) | 14.65(8.04to22.83) | 0.34 (0.07 to 0.61) |
| Belize | 8.8(4.33to14.09) | 43.14(26to63.49) | 8.77(4.35to14.05) | 13.18(7.92to19.43) | 1.23 (1.09 to 1.37) |
| Benin | 30.83(13.43to55.21) | 247.52(124.32to406.91) | 1.44(0.63to2.6) | 4.25(2.15to6.97) | 3.87 (3.54 to 4.2) |
| Bermuda | 18.28(10.87to26.6) | 19.08(11.03to28.47) | 28.27(16.83to41) | 16.45(9.49to24.63) | -1.77 (-2.3 to -1.24) |
| Bhutan | 2.17(0.51to5.56) | 18.26(6.77to34.6) | 0.75(0.17to1.96) | 3.01(1.1to5.7) | 5.15 (4.88 to 5.42) |
| Bolivia (Plurinational State of) | 178.28(86.2to308.43) | 1141.39(622.73to1834.23) | 5.04(2.42to8.69) | 12.23(6.68to19.52) | 3.04 (2.92 to 3.15) |
| Bosnia and Herzegovina | 386.57(204.45to601.01) | 1191(664.33to1917.12) | 8.66(4.62to13.44) | 20.32(11.32to32.68) | 3.37 (3.05 to 3.7) |
| Botswana | 14.1(6.11to27.05) | 132.52(73.46to210.37) | 2.22(0.95to4.21) | 8.23(4.69to12.93) | 4.11 (3.72 to 4.51) |
| Brazil | 5388.08(2790.85to8674.5) | 26918.34(16515.43to38620.14) | 5.49(2.85to8.87) | 11.07(6.78to15.9) | 2.64 (2.44 to 2.83) |
| Brunei Darussalam | 4.97(1.33to11.02) | 35.99(16.93to61.03) | 4.47(1.2to9.84) | 10.69(4.96to17.86) | 3.82 (3.55 to 4.09) |
| Bulgaria | 872.84(507.3to1265.07) | 2534.44(1425.05to3990.96) | 6.97(4.03to10.12) | 20.32(11.52to32.22) | 6.1 (5.18 to 7.03) |
| Burkina Faso | 30.84(8.63to69.11) | 255.51(116.26to458.62) | 0.65(0.18to1.46) | 2.37(1.09to4.19) | 4.64 (4.57 to 4.7) |
| Burundi | 23.02(6.83to50.63) | 76.76(27.29to157.54) | 0.9(0.26to2) | 1.44(0.51to2.97) | 1.54 (1.41 to 1.67) |
| Cabo Verde | 2.6(1.12to4.58) | 49.63(27.19to78.49) | 1.2(0.53to2.12) | 10.54(5.74to16.63) | 7.31 (6.63 to 7.99) |
| Cambodia | 30.28(6.7to77.85) | 278.75(117.54to506.47) | 0.59(0.13to1.52) | 2.11(0.89to3.87) | 4.89 (4.64 to 5.14) |
| Cameroon | 124.41(64.78to204.68) | 566.55(284.39to914.34) | 2.46(1.28to4.05) | 4.05(2.08to6.51) | 1.62 (1.53 to 1.72) |
| Canada | 3878.1(2134.15to6000.22) | 11517.02(6783.54to16786.44) | 12.32(6.79to19.04) | 17.94(10.53to25.89) | 1.53 (1.2 to 1.87) |
| Central African Republic | 11.3(3.23to25.16) | 33.26(11.54to70.66) | 0.82(0.23to1.85) | 1.25(0.42to2.71) | 1.74 (1.56 to 1.93) |
| Chad | 13.19(3.7to29.25) | 88.55(38.13to162.7) | 0.45(0.13to1) | 1.35(0.59to2.49) | 3.92 (3.83 to 4.01) |
| Chile | 2070.54(1073.52to3266.78) | 5907.08(3431.03to8864.07) | 19.57(10.13to30.72) | 24.69(14.3to36.96) | 1.15 (1.03 to 1.26) |
| China | 8117.04(1905.54to18423.87) | 70543.6(29312.42to127228.96) | 0.86(0.2to1.95) | 3.45(1.44to6.26) | 5.71 (5.23 to 6.19) |
| Colombia | 781.51(395.91to1278.83) | 3819.04(2011.48to6321.07) | 4.03(2.05to6.58) | 7.23(3.82to11.98) | 2.02 (1.89 to 2.15) |
| Comoros | 3.85(1.13to8.18) | 19.87(9.52to34.5) | 1.63(0.51to3.45) | 3.78(1.81to6.61) | 2.86 (2.7 to 3.02) |
| Congo | 36.55(14.58to69.16) | 193(94.94to345.8) | 3.03(1.22to5.68) | 6.12(3.05to10.67) | 2.36 (2.19 to 2.54) |
| Cook Islands | 0.54(0.3to0.85) | 1.29(0.77to1.94) | 3.84(2.09to6.05) | 5.51(3.33to8.35) | 0.99 (0.85 to 1.13) |
| Costa Rica | 109.02(58.36to171.46) | 631.1(324.91to1050.25) | 5.93(3.18to9.3) | 12.03(6.2to20.03) | 2.7 (2.51 to 2.88) |
| Croatia | 507.34(273.68to767.57) | 2161.54(1203.44to3304.04) | 7.74(4.2to11.67) | 26.58(14.89to40.61) | 4.5 (3.85 to 5.16) |
| Cuba | 1337.61(777.32to2007.75) | 1980.61(1108.02to3126.95) | 12.91(7.46to19.36) | 11.15(6.26to17.57) | -0.36 (-1.02 to 0.31) |
| Cyprus | 25.47(12.08to43.84) | 138.62(74.37to218.53) | 3.09(1.45to5.33) | 7.28(3.91to11.49) | 3.57 (3.2 to 3.95) |
| Czechia | 3858.82(2216.07to5697.53) | 8080.14(4752.94to12078.81) | 28.64(16.41to42.49) | 40.4(23.51to60.07) | 0.89 (0.4 to 1.38) |
| C么te d'Ivoire | 98.95(45.05to181.29) | 521.9(262.19to911.02) | 1.97(0.87to3.64) | 3.85(1.93to6.71) | 2.08 (1.96 to 2.19) |
| Democratic People's Republic of Korea | 167.42(29.43to432) | 383.53(72.75to974.09) | 0.92(0.16to2.36) | 1.16(0.22to2.93) | 0.83 (0.79 to 0.88) |
| Democratic Republic of the Congo | 288.49(120.68to538.55) | 710.86(294.1to1351.36) | 1.6(0.65to3) | 1.71(0.7to3.28) | -0.62 (-1.33 to 0.09) |
| Denmark | 734.1(385.49to1159.62) | 1789.75(997.66to2827.72) | 9.74(5.11to15.35) | 17.11(9.53to26.84) | 1.22 (0.62 to 1.83) |
| Djibouti | 1.44(0.31to3.61) | 30.26(12.92to56.88) | 0.83(0.17to2.07) | 4.22(1.84to7.9) | 6.52 (6.22 to 6.82) |
| Dominica | 9.15(5.01to14.05) | 13.3(7.42to20.84) | 14.28(7.83to21.65) | 15.5(8.64to24.22) | 0.42 (0.1 to 0.74) |
| Dominican Republic | 221.29(98.42to389.83) | 1399.34(708.31to2442.23) | 5.02(2.28to8.83) | 13.89(7.01to24.29) | 4.22 (3.48 to 4.96) |
| Ecuador | 471.85(287.97to690) | 2123.93(1216.17to3222.93) | 7.88(4.76to11.48) | 13.45(7.7to20.37) | 2.29 (1.88 to 2.69) |
| Egypt | 1033.96(589.58to1555.34) | 5731.68(3139.87to9670.85) | 3.07(1.75to4.62) | 7.69(4.21to13.09) | 3.02 (2.83 to 3.21) |
| El Salvador | 129.2(65.88to207.37) | 495.55(258.94to813.32) | 4.06(2.07to6.48) | 8.46(4.4to13.87) | 2.49 (2.25 to 2.74) |
| Equatorial Guinea | 1.6(0.34to4.02) | 50.82(22.11to102) | 0.71(0.15to1.81) | 8.86(4.01to17.69) | 11.11 (10.22 to 12) |
| Eritrea | 6.85(2.04to15.57) | 76.88(32.84to144.97) | 0.61(0.19to1.37) | 2.67(1.16to4.88) | 5.1 (4.52 to 5.69) |
| Estonia | 209.83(117.44to322.2) | 843.95(497.62to1296.44) | 10.29(5.78to15.78) | 34.86(20.66to53.86) | 4.38 (3.59 to 5.18) |
| Eswatini | 17.04(8.77to27.77) | 73.93(36.78to126.82) | 5.21(2.76to8.52) | 11.42(5.82to19.52) | 2.42 (1.59 to 3.26) |
| Ethiopia | 208.82(41.71to584.59) | 1178.6(460.45to2283.99) | 0.93(0.18to2.68) | 2.57(0.96to5.05) | 3.71 (3.15 to 4.26) |
| Fiji | 14.26(7.65to22.88) | 40.81(23.72to63.36) | 3.19(1.66to5.11) | 4.9(2.83to7.57) | 1.1 (0.88 to 1.32) |
| Finland | 1177.74(635.98to1845.84) | 1954.34(1090.36to2971.17) | 17.14(9.21to26.94) | 17.62(9.95to26.71) | -0.08 (-0.21 to 0.04) |
| France | 10718.71(5594.36to17166.48) | 19143.16(10708.74to29507.31) | 13.97(7.26to22.37) | 16.01(9.01to24.72) | 0.42 (0.36 to 0.48) |
| Gabon | 20.51(8.28to45.3) | 131.33(64.97to236.6) | 3.38(1.35to7.51) | 10.89(5.38to19.64) | 3.85 (3.54 to 4.16) |
| Gambia | 4.1(1.62to7.95) | 34.61(17.11to61.09) | 1.04(0.41to2.01) | 3.25(1.6to5.72) | 3.92 (3.7 to 4.13) |
| Georgia | 706.96(378.23to1173.51) | 968.05(534.6to1467.63) | 11.02(5.93to18.37) | 17.79(9.89to27.12) | 2.25 (1.72 to 2.78) |
| Germany | 21198.92(11588.88to32952.3) | 32878.97(18253.97to49440.79) | 17.82(9.7to27.77) | 18.9(10.63to28.41) | -0.15 (-0.27 to -0.02) |
| Ghana | 146.78(59.57to272.49) | 952.25(527.21to1558.1) | 1.86(0.75to3.48) | 4.98(2.81to8.05) | 2.44 (1.91 to 2.96) |
| Greece | 1706.96(881.82to2709.74) | 2964.25(1598.89to4563.27) | 11.41(5.88to18.16) | 14.62(7.93to22.62) | 0.51 (0.3 to 0.73) |
| Greenland | 7.56(4.08to11.9) | 26.53(14.76to41.26) | 19.21(10.41to30.01) | 35.47(19.57to55.01) | 1.9 (1.53 to 2.27) |
| Grenada | 4.48(2.2to7.51) | 12.55(7.34to18.84) | 7(3.5to11.61) | 10.52(6.16to15.79) | 1.21 (0.67 to 1.75) |
| Guam | 8.21(4.1to13.1) | 17.27(9.42to27.01) | 9.02(4.44to14.55) | 9.08(4.98to14.09) | 0.15 (-0.22 to 0.52) |
| Guatemala | 123.31(44.23to231.29) | 834.2(422.69to1414.75) | 2.81(1.02to5.29) | 6.9(3.51to11.66) | 3.49 (2.93 to 4.05) |
| Guinea | 35.22(14.53to66.83) | 127.69(63.22to216.94) | 1(0.41to1.9) | 2.1(1.03to3.56) | 2.58 (2.55 to 2.61) |
| Guinea-Bissau | 5.47(1.72to11.68) | 23.38(10.58to41.45) | 1.19(0.38to2.56) | 2.64(1.19to4.74) | 2.66 (2.51 to 2.81) |
| Guyana | 44.34(21.61to75.98) | 87.12(46.56to144.16) | 9.85(4.79to16.79) | 12.05(6.42to19.83) | 0.78 (0.53 to 1.03) |
| Haiti | 97.04(35.07to191.24) | 239.22(95.55to479.28) | 2.57(0.92to5.15) | 2.86(1.14to5.79) | 0.74 (0.44 to 1.04) |
| Honduras | 57.75(23.35to103.76) | 481.72(238.88to899.51) | 2.6(1.07to4.63) | 7.61(3.8to14.2) | 3.93 (3.62 to 4.24) |
| Hungary | 4091.97(2490.71to5946.17) | 5677.4(3466.16to8528.51) | 28.45(17.34to41.34) | 32.14(19.58to48.24) | 0.23 (-0.12 to 0.57) |
| Iceland | 63.8(33.66to97.81) | 129.51(70.64to198.52) | 23.33(12.27to35.77) | 25.08(13.66to38.59) | 0.26 (0.07 to 0.44) |
| India | 2624.18(971.74to5492.98) | 26141.41(14120.53to42446.07) | 0.51(0.19to1.06) | 2.13(1.14to3.47) | 5.07 (4.9 to 5.24) |
| Indonesia | 898.52(257.82to1924.7) | 10492.48(5065.5to19918.9) | 0.75(0.21to1.64) | 4.09(1.95to7.86) | 6.3 (6.16 to 6.45) |
| Iran (Islamic Republic of) | 902.43(445.01to1506.6) | 5319.29(3269.13to7727.75) | 3.06(1.48to5.11) | 6.73(4.14to9.77) | 2.79 (2.6 to 2.97) |
| Iraq | 614.43(298to1036.32) | 3127.91(1740.39to5062.05) | 7.18(3.43to12.08) | 11.68(6.49to18.53) | 1.95 (1.61 to 2.29) |
| Ireland | 504.42(261.19to798.01) | 1299.03(725.32to1989.55) | 12.87(6.64to20.45) | 18.19(10.22to27.91) | 1.26 (0.96 to 1.56) |
| Israel | 549.35(289.31to872.51) | 1484.57(795.26to2295.61) | 11.51(6.04to18.24) | 13.43(7.25to20.86) | 0.13 (-0.21 to 0.48) |
| Italy | 11461.28(6007.23to18321.55) | 17687.85(9608.82to27537.28) | 13.45(7.01to21.53) | 13.92(7.45to21.73) | -0.08 (-0.17 to 0.01) |
| Jamaica | 136.55(77.92to211.9) | 245.95(141.21to389.55) | 7.98(4.57to12.34) | 8.21(4.72to13.05) | 0.45 (0.02 to 0.87) |
| Japan | 5587.82(1874.16to10987.36) | 10989.12(3903.37to21030.57) | 3.25(1.09to6.4) | 3.65(1.32to6.97) | 0.21 (0.04 to 0.37) |
| Jordan | 68.69(38.54to104.41) | 750.27(447.55to1071.82) | 4.4(2.5to6.74) | 9.96(5.97to14.15) | 3.33 (3.1 to 3.56) |
| Kazakhstan | 2181.25(1124.69to3632.07) | 4353.13(2606.07to6295.75) | 16.14(8.33to26.63) | 23.02(13.8to33.23) | 0.77 (0.36 to 1.18) |
| Kenya | 80.58(31.3to152.51) | 840.4(463.94to1340.86) | 0.84(0.32to1.61) | 3.22(1.73to5.18) | 5.44 (5.13 to 5.74) |
| Kiribati | 4.74(2.24to7.91) | 11.76(5.56to21.01) | 10.34(4.79to17.43) | 13.07(6.02to23.45) | 0.22 (-0.26 to 0.7) |
| Kuwait | 58.96(35.93to85.76) | 339.75(207.49to499.63) | 7.33(4.37to10.88) | 10.64(6.37to15.82) | 1.02 (0.44 to 1.6) |
| Kyrgyzstan | 138.62(71.9to224.84) | 523.06(287.66to820.31) | 4.39(2.3to7.07) | 10.01(5.47to15.76) | 1.92 (1.44 to 2.41) |
| Lao People's Democratic Republic | 18.32(4.36to45.4) | 153.72(69.51to270.54) | 0.78(0.18to1.96) | 2.97(1.35to5.2) | 5.04 (4.91 to 5.17) |
| Latvia | 316.16(171.72to484.39) | 1169.5(685.62to1783.18) | 8.91(4.85to13.59) | 32.42(19.11to49.41) | 4.2 (3.45 to 4.95) |
| Lebanon | 138.9(69.07to237.66) | 718.57(379.57to1179.01) | 5.68(2.85to9.72) | 13.86(7.31to22.8) | 3.82 (3.53 to 4.12) |
| Lesotho | 15.86(6.19to30.44) | 85.63(44.59to141.38) | 1.51(0.59to2.87) | 6.1(3.2to9.98) | 5.44 (5.15 to 5.74) |
| Liberia | 24.1(11.45to40.48) | 110.73(52.96to192.5) | 2.05(0.96to3.44) | 4.27(2.06to7.65) | 3.95 (3.08 to 4.82) |
| Libya | 155.24(77.07to265.93) | 816.22(432.93to1292.63) | 7.72(3.79to13.1) | 14.6(7.73to22.95) | 2.39 (2.19 to 2.6) |
| Lithuania | 468.01(251.15to735.31) | 1735.51(952.4to2637.12) | 10.42(5.63to16.33) | 33.19(18.04to50.61) | 4 (3.21 to 4.79) |
| Luxembourg | 47.55(26.13to73.51) | 73.02(41.35to114.73) | 8.98(4.93to13.89) | 7.64(4.32to11.99) | -0.69 (-0.78 to -0.6) |
| Madagascar | 48.21(17.44to97.03) | 266.25(121.6to475.73) | 0.85(0.31to1.69) | 2.07(0.95to3.67) | 3.77 (3.45 to 4.09) |
| Malawi | 98.98(25.86to230.65) | 559.3(252.04to958.65) | 2.24(0.58to5.23) | 6.94(3.19to12.03) | 4.52 (4.23 to 4.8) |
| Malaysia | 233.96(109.19to414.13) | 1891.52(1019.03to3152.71) | 2.31(1.05to4.12) | 6.6(3.56to10.98) | 3.61 (3.45 to 3.77) |
| Maldives | 0.89(0.2to2.26) | 10.48(5.14to17.86) | 0.82(0.18to2.13) | 2.77(1.32to4.81) | 4.29 (4.11 to 4.47) |
| Mali | 35.1(11.24to71) | 209.62(101.02to362.56) | 0.78(0.25to1.57) | 2.17(1.04to3.75) | 3.67 (3.55 to 3.79) |
| Malta | 41.29(20.07to68.34) | 100.9(52.03to160.98) | 9.59(4.66to15.92) | 12(6.22to19.3) | 1.03 (0.78 to 1.28) |
| Marshall Islands | 0.42(0.15to0.82) | 1.74(0.78to3.06) | 2.19(0.78to4.3) | 3.87(1.7to6.89) | 1.52 (1.24 to 1.81) |
| Mauritania | 30.24(15.09to53.06) | 115.37(62.34to186.97) | 2.87(1.44to5.04) | 5.06(2.79to8.11) | 1.98 (1.91 to 2.05) |
| Mauritius | 18.96(9.93to30.58) | 106.26(58.79to169.82) | 2.32(1.21to3.77) | 6.02(3.34to9.6) | 3.32 (3.03 to 3.62) |
| Mexico | 4992.67(2787.25to7536) | 22665.56(13075.42to33444.28) | 10.62(5.91to16.06) | 18.46(10.66to27.29) | 1.84 (1.74 to 1.95) |
| Micronesia (Federated States of) | 2.48(1.24to4.14) | 5.66(2.79to10.04) | 4.57(2.28to7.76) | 6.39(3.16to11.27) | 0.77 (0.44 to 1.1) |
| Monaco | 10.5(5.35to17.22) | 19.44(10.32to30.26) | 17.22(8.87to28.13) | 23.7(12.58to36.53) | 1.23 (1.03 to 1.43) |
| Mongolia | 49.84(24.28to84.48) | 321.57(170.86to517.92) | 4.6(2.25to7.82) | 12.02(6.36to19.44) | 3.71 (3.49 to 3.92) |
| Montenegro | 95.24(55.04to141.19) | 216.41(129.97to324.16) | 14.77(8.54to21.78) | 22.24(13.34to33.26) | 1.78 (1.66 to 1.9) |
| Morocco | 281.62(142.43to464.33) | 1652.44(847.77to2656.63) | 1.84(0.94to3.06) | 4.8(2.49to7.71) | 3.29 (3.11 to 3.46) |
| Mozambique | 33.1(7.66to78.69) | 307.83(129.93to581.26) | 0.48(0.11to1.14) | 2.32(0.98to4.44) | 6.48 (6.2 to 6.76) |
| Myanmar | 163.32(33.13to429.89) | 1418.11(656.7to2470.01) | 0.62(0.12to1.65) | 2.76(1.26to4.8) | 5.98 (5.69 to 6.27) |
| Namibia | 25.59(13.6to41.88) | 119.32(67.5to190.26) | 3.38(1.81to5.51) | 7.98(4.58to12.64) | 2.94 (2.71 to 3.17) |
| Nauru | 0.38(0.19to0.76) | 0.52(0.26to0.87) | 7.14(3.45to13.71) | 7.99(4.04to13.35) | -0.39 (-0.72 to -0.05) |
| Nepal | 32.2(7.13to84.87) | 449.45(165.73to922.83) | 0.3(0.06to0.8) | 1.86(0.68to3.83) | 7.11 (6.83 to 7.39) |
| Netherlands | 2750.59(1393.89to4385.51) | 5690.35(2973.89to9048.7) | 14.4(7.27to23.02) | 17.86(9.41to28.09) | 0.6 (0.44 to 0.75) |
| New Zealand | 578.5(315.85to901.8) | 1361.68(793.19to2012.77) | 15.17(8.32to23.66) | 18.91(11.13to27.96) | 0.91 (0.78 to 1.05) |
| Nicaragua | 51.03(23.31to89.95) | 376.8(195.81to611.84) | 2.95(1.34to5.2) | 7.98(4.15to12.99) | 3.51 (3.13 to 3.88) |
| Niger | 23.48(8.82to46.72) | 115.66(51.67to208.95) | 0.72(0.27to1.43) | 1.28(0.57to2.29) | 1.94 (1.83 to 2.05) |
| Nigeria | 518.56(204.45to1020.07) | 3052.32(1516.36to5110.63) | 1.06(0.42to2.09) | 2.95(1.47to4.93) | 3.64 (3.54 to 3.74) |
| Niue | 0.11(0.06to0.18) | 0.17(0.09to0.28) | 5.19(2.71to8.48) | 8.06(4.38to13.23) | 1.26 (0.99 to 1.54) |
| North Macedonia | 81.68(44.86to126.73) | 444.04(254.35to706.59) | 4.12(2.26to6.4) | 13.69(7.89to21.65) | 5.22 (4.64 to 5.8) |
| Northern Mariana Islands | 3.27(1.73to5.47) | 6.59(3.69to9.84) | 11.59(6.13to18.86) | 10.95(6.15to16.31) | -0.6 (-1.21 to 0.03) |
| Norway | 755.28(389.59to1200.64) | 1330.13(719.62to2080.8) | 12.2(6.3to19.41) | 14.87(8.09to23.19) | 0.8 (0.59 to 1) |
| Oman | 18.9(8.45to33.36) | 201.34(119.13to303.23) | 2.25(0.99to4.01) | 8.85(5.35to13.11) | 5.21 (4.59 to 5.84) |
| Pakistan | 552.08(139.41to1248.73) | 4570.24(2149.51to7930.73) | 0.91(0.23to2.06) | 3.65(1.73to6.39) | 5.36 (4.93 to 5.79) |
| Palau | 0.37(0.2to0.61) | 1.28(0.68to1.98) | 3.32(1.79to5.4) | 5.1(2.75to7.9) | 1.12 (0.81 to 1.43) |
| Palestine | 41.06(17.53to74.8) | 214.59(119.09to331.39) | 4.35(1.84to7.93) | 7.79(4.28to12.08) | 1.69 (1.38 to 1.99) |
| Panama | 44.16(15.32to82.14) | 406.7(213.49to680.06) | 2.82(0.98to5.24) | 9.77(5.14to16.31) | 5.19 (4.97 to 5.41) |
| Papua New Guinea | 18.32(5.64to40.35) | 74.65(27.56to155.55) | 0.81(0.24to1.82) | 1.2(0.43to2.5) | 0.88 (0.63 to 1.13) |
| Paraguay | 149.72(77.81to244.36) | 557.34(292.34to931.88) | 6.15(3.2to10.05) | 9.41(4.9to15.68) | 1.27 (1.14 to 1.41) |
| Peru | 1065.77(516.86to1806.73) | 3753.8(1931.15to6229.7) | 8.13(3.97to13.78) | 11.54(5.96to19.1) | 1.07 (0.74 to 1.4) |
| Philippines | 675.39(287.13to1220.9) | 3701.85(1890.44to6068.85) | 1.87(0.79to3.4) | 4.11(2.08to6.76) | 2.33 (2.05 to 2.6) |
| Poland | 4265.89(2378.16to6391.79) | 22559.36(13062.05to33281.56) | 9.65(5.35to14.48) | 33.8(19.58to49.89) | 4.34 (3.3 to 5.4) |
| Portugal | 939.02(452.23to1541.25) | 1878.56(958.68to3030.07) | 7.01(3.37to11.53) | 9.13(4.63to14.7) | 0.69 (0.46 to 0.92) |
| Puerto Rico | 479.95(279.91to705.07) | 811.29(477.99to1232.19) | 13.34(7.78to19.56) | 13.84(8.25to21.04) | 0.48 (0.21 to 0.74) |
| Qatar | 20.63(11.25to33.22) | 239.74(135.02to375.17) | 15.08(7.81to24.37) | 23.67(12.87to36.77) | 1.35 (1.02 to 1.69) |
| Republic of Korea | 626.58(195.11to1264.78) | 3883.93(1594.27to6900.57) | 1.87(0.59to3.77) | 4.37(1.79to7.75) | 2.59 (1.92 to 3.26) |
| Republic of Moldova | 406.16(223.29to633.45) | 1054.19(625.51to1558.56) | 8.63(4.77to13.51) | 18.34(10.87to27.24) | 3.38 (2.87 to 3.89) |
| Romania | 2831.3(1736.86to4088.75) | 7112.73(4467.3to10565.83) | 9.86(6.01to14.27) | 21.66(13.54to32.09) | 2.83 (2.7 to 2.95) |
| Russian Federation | 36055.09(21423.18to53404.99) | 66344.98(39557.99to96112.31) | 19.47(11.56to28.92) | 28.68(17.12to41.65) | 1.04 (0.79 to 1.28) |
| Rwanda | 40.8(10.87to90.18) | 216.09(95.92to381.86) | 1.27(0.33to2.81) | 3.22(1.42to5.67) | 3.38 (2.85 to 3.9) |
| Saint Kitts and Nevis | 8.88(5.03to13.8) | 12.42(6.81to19.33) | 26.16(14.84to40.45) | 16.69(9.09to26.1) | -1.69 (-2.3 to -1.07) |
| Saint Lucia | 8.77(4.56to14.04) | 20.19(11.33to31.49) | 9.77(5.13to15.63) | 9.18(5.1to14.27) | -0.49 (-0.93 to -0.05) |
| Saint Vincent and the Grenadines | 6.83(3.36to11.4) | 14.8(8.6to22.37) | 9.49(4.73to15.77) | 10.85(6.3to16.44) | 0.73 (0.26 to 1.19) |
| Samoa | 4.85(2.72to7.47) | 8.62(4.6to13.77) | 5.15(2.89to7.96) | 5.5(2.92to8.78) | -0.23 (-0.45 to -0.02) |
| San Marino | 2.78(1.45to4.44) | 6.41(3.07to11.4) | 8.63(4.52to13.75) | 11.24(5.4to20.08) | 1.19 (1.08 to 1.3) |
| Sao Tome and Principe | 0.97(0.4to1.76) | 5.61(2.81to10.12) | 1.41(0.58to2.55) | 4.55(2.25to8.32) | 4.23 (4.16 to 4.3) |
| Saudi Arabia | 236.67(116.51to398.29) | 3114.53(1787.82to4881.27) | 3.31(1.63to5.6) | 12.26(7.08to18.68) | 4.19 (3.86 to 4.53) |
| Senegal | 55.45(24.93to97.16) | 280.94(144.63to466.53) | 1.55(0.69to2.74) | 3.31(1.72to5.44) | 2.72 (2.54 to 2.9) |
| Serbia | 1784.49(961.33to2721.38) | 3840.74(2166.32to5856.79) | 14.99(8.15to23.07) | 25.64(14.42to39.42) | 2.01 (1.89 to 2.12) |
| Seychelles | 2.2(1.15to3.57) | 9.83(5.49to15.36) | 3.95(2.07to6.39) | 8.02(4.43to12.58) | 1.4 (0.86 to 1.94) |
| Sierra Leone | 16.21(4.78to35.25) | 84.01(37.2to152.91) | 0.81(0.24to1.75) | 2.03(0.9to3.72) | 3.3 (3 to 3.61) |
| Singapore | 83.31(26.04to164.06) | 511.14(267.53to821.72) | 3.43(1.06to6.75) | 6.3(3.28to10.17) | 1.99 (1.67 to 2.32) |
| Slovakia | 997.92(576.34to1478.94) | 2941.05(1715.96to4488.3) | 16.7(9.65to24.76) | 32.32(18.9to49.42) | 2.07 (1.63 to 2.52) |
| Slovenia | 322.7(176.01to506.83) | 960(534.48to1521.83) | 13.17(7.18to20.71) | 24.08(13.11to38.7) | 2.29 (1.9 to 2.69) |
| Solomon Islands | 3.59(1.24to7.47) | 15.5(6.85to29.36) | 2.08(0.68to4.37) | 3.62(1.56to6.91) | 1.46 (0.94 to 1.98) |
| Somalia | 17.3(3.29to50.39) | 61.92(10.27to176.39) | 0.56(0.1to1.66) | 0.78(0.13to2.23) | 1.62 (1.47 to 1.77) |
| South Africa | 1285.15(809.88to1865.16) | 3872.34(2538.57to5366.48) | 5.53(3.47to8.02) | 8.1(5.29to11.24) | 1.49 (1.31 to 1.67) |
| South Sudan | 50.48(16.43to131.53) | 172.33(76.55to360.97) | 1.94(0.63to5.19) | 3.96(1.77to8.27) | 2.83 (2.69 to 2.98) |
| Spain | 5462.96(2950.13to8548.18) | 12767.72(7239.85to19607.68) | 10.43(5.59to16.32) | 15.16(8.55to23.28) | 1.07 (0.9 to 1.25) |
| Sri Lanka | 658.04(293.97to1142.57) | 2162.15(1053.01to3712.06) | 5.53(2.44to9.65) | 8.15(3.96to13.91) | -0.43 (-1.35 to 0.5) |
| Sudan | 143.39(53.86to298.15) | 1229.29(533.5to2452.24) | 1.39(0.51to2.86) | 5.69(2.44to11.48) | 5.31 (4.94 to 5.68) |
| Suriname | 25.23(12.92to39.7) | 63.79(35.21to102.12) | 8.77(4.56to13.85) | 10.06(5.58to16.04) | 0.67 (0.14 to 1.21) |
| Sweden | 2691.95(1379.32to4295.59) | 3189.63(1754.44to4954.57) | 19.83(10.18to31.69) | 16.73(9.34to25.93) | -0.78 (-0.89 to -0.66) |
| Switzerland | 656.91(344.86to1039.55) | 1654.06(889.73to2586.02) | 6.69(3.51to10.63) | 10.2(5.48to15.99) | 0.71 (0.21 to 1.23) |
| Syrian Arab Republic | 124.37(64.05to206.46) | 541.47(287.94to868.5) | 2.09(1.07to3.47) | 3.91(2.09to6.28) | 2.16 (1.96 to 2.36) |
| Taiwan (Province of China) | 406.73(171.22to719.52) | 3852.28(1756.93to6639.68) | 2.39(1.01to4.2) | 9.93(4.54to17.11) | 4.57 (3.87 to 5.28) |
| Tajikistan | 122.12(45.27to255.04) | 408.9(179.7to726.1) | 3.99(1.48to8.18) | 6.34(2.8to11.3) | 1.44 (0.78 to 2.11) |
| Thailand | 458.68(159to896.75) | 3359.15(1618.12to5761.63) | 1.1(0.37to2.19) | 3.27(1.57to5.58) | 2.96 (2.47 to 3.45) |
| Timor-Leste | 1.6(0.34to4.17) | 10.65(3.22to23.5) | 0.44(0.09to1.18) | 1.21(0.37to2.68) | 4.39 (3.7 to 5.09) |
| Togo | 16.52(6.47to31.66) | 129.06(65.82to216.72) | 1.16(0.45to2.22) | 2.9(1.48to4.84) | 2.95 (2.83 to 3.08) |
| Tokelau | 0.03(0.01to0.06) | 0.07(0.03to0.11) | 2.68(1.15to4.8) | 4.85(2.52to8.17) | 1.87 (1.69 to 2.04) |
| Tonga | 2.1(1.1to3.5) | 4.6(2.39to7.59) | 3.4(1.76to5.71) | 5.55(2.9to9.12) | 1.05 (0.43 to 1.68) |
| Trinidad and Tobago | 171.41(103.76to253.01) | 225.24(127.77to361.47) | 18.74(11.19to27.73) | 12.35(7to19.69) | -1.83 (-2.48 to -1.17) |
| Tunisia | 159.78(82.78to268.6) | 865.14(449.62to1440.38) | 2.96(1.54to4.99) | 6.61(3.46to10.99) | 2.82 (2.76 to 2.87) |
| Turkey | 3300.37(1757.26to5371.03) | 12189.11(6870.95to18424.57) | 8.34(4.41to13.44) | 13.3(7.5to20.12) | 1.44 (1.33 to 1.54) |
| Turkmenistan | 152.35(78.22to248.16) | 1183.14(657.69to1800.14) | 7.14(3.68to11.59) | 25.47(14.1to38.75) | 5.36 (4.89 to 5.83) |
| Tuvalu | 0.18(0.07to0.35) | 0.47(0.22to0.84) | 2.47(0.96to4.61) | 4.35(2.01to7.8) | 1.37 (1.03 to 1.71) |
| Uganda | 52.15(13.55to116.53) | 694.1(337.04to1202.67) | 0.73(0.19to1.65) | 4.37(2.08to7.5) | 7.13 (6.84 to 7.42) |
| Ukraine | 13365.89(7480.1to20976.38) | 21109.21(12685.04to32555.31) | 18.54(10.39to28.9) | 29.42(17.61to45.61) | 1.4 (1.15 to 1.65) |
| United Arab Emirates | 126.14(43.61to318.26) | 3180.57(1117.73to5899.57) | 16.01(4.84to43.1) | 39.6(12.26to74.67) | 3.16 (2.89 to 3.43) |
| United Kingdom | 13404(7140.18to20737.92) | 23486.91(13516.38to34794.99) | 16.19(8.59to25.06) | 20.69(11.95to30.57) | 0.68 (0.54 to 0.82) |
| United Republic of Tanzania | 213.33(89.27to403.31) | 1395.32(727.16to2353.15) | 1.78(0.75to3.36) | 5.09(2.66to8.64) | 4.03 (3.74 to 4.32) |
| United States of America | 59382.15(33842.22to88131.4) | 120883.1(74181.04to168531.12) | 20.12(11.52to29.74) | 23.09(14.17to32.2) | 0.22 (0.07 to 0.36) |
| United States Virgin Islands | 16.82(9.68to25.33) | 47.48(27.26to71.17) | 17.53(10.08to26.48) | 27.66(15.99to42.1) | 1.84 (1.55 to 2.13) |
| Uruguay | 982.01(494.17to1576.17) | 1625.97(882.32to2483.47) | 26.45(13.29to42.62) | 34.25(18.51to52.13) | 0.65 (0.51 to 0.8) |
| Uzbekistan | 488.2(213.98to876.87) | 3017.74(1702.47to4695.43) | 3.89(1.71to7.02) | 11.09(6.3to17.11) | 3.8 (3.68 to 3.92) |
| Vanuatu | 1.5(0.58to3) | 6.78(3.14to12.67) | 1.92(0.72to3.91) | 3.36(1.53to6.34) | 1.69 (1.53 to 1.85) |
| Venezuela (Bolivarian Republic of) | 1471.62(784.66to2308.4) | 4366.89(2242.42to7426.73) | 13.32(7.09to20.69) | 14.24(7.28to24.15) | 0.47 (-0.05 to 0.99) |
| Viet Nam | 149.66(31.6to380.34) | 1754.45(687.53to3330.27) | 0.36(0.08to0.9) | 1.72(0.68to3.29) | 6.42 (6.08 to 6.75) |
| Yemen | 48.73(14.15to110.93) | 336.37(154.18to591.21) | 0.87(0.24to1.97) | 2.17(1to3.82) | 4.09 (3.81 to 4.37) |
| Zambia | 65.72(22.46to134.44) | 455.78(215.94to824.64) | 1.99(0.67to4.07) | 5.8(2.72to10.37) | 3.64 (3.38 to 3.91) |
| Zimbabwe | 86.4(45.54to140.85) | 284.26(147.59to458.83) | 1.88(0.98to3.08) | 3.56(1.86to5.71) | 2.08 (1.64 to 2.52) |

ASDR, age-standard DALYs rate; DALYs, disability-adjusted life years; EAPC, estimated annual percentage change.
